# Novel Immune Modulators Enhance *Caenorhabditis elegans* Resistance to Multiple Pathogens

**DOI:** 10.1128/mSphere.00950-20

**Published:** 2021-01-06

**Authors:** Nicholas A. Hummell, Alexey V. Revtovich, Natalia V. Kirienko

**Affiliations:** a Department of BioSciences, Rice University, Houston, Texas, USA; University of Rochester

**Keywords:** *C. elegans*, *E. faecalis*, MDT-15/MED15, NHR-49/HNF4, *P. aeruginosa*, PMK-1/p38 MAPK, *S. aureus*, high-throughput screen, immune modulators

## Abstract

Traditionally, treatments for bacterial infection have focused on killing the microbe or preventing its growth. As antimicrobial resistance becomes more ubiquitous, the feasibility of this approach is beginning to wane and attention has begun to shift toward disrupting the host-pathogen interaction by improving the host defense. Using a high-throughput, fragment-based screen to identify compounds that alleviate Pseudomonas aeruginosa-mediated killing of Caenorhabditis elegans, we identified over 20 compounds that stimulated host defense gene expression. Five of these molecules were selected for further characterization. Four of five compounds showed little toxicity against mammalian cells or worms, consistent with their identification in a phenotypic, high-content screen. Each of the compounds activated several host defense pathways, but the pathways were generally dispensable for compound-mediated rescue in liquid killing, suggesting redundancy or that the activation of unknown pathway(s) may be driving compound effects. A genetic mechanism was identified for LK56, which required the Mediator subunit MDT-15/MED15 and NHR-49/HNF4 for its function. Interestingly, LK32, LK34, LK38, and LK56 also rescued C. elegans from P. aeruginosa in an agar-based assay, which uses different virulence factors and defense mechanisms. Rescue in an agar-based assay for LK38 entirely depended upon the PMK-1/p38 MAPK pathway. Three compounds—LK32, LK34, and LK56—also conferred resistance to Enterococcus faecalis, and the two lattermost, LK34 and LK56, also reduced pathogenesis from Staphylococcus aureus. This study supports a growing role for MDT-15 and NHR-49 in immune response and identifies five molecules that have significant potential for use as tools in the investigation of innate immunity.

**IMPORTANCE** Trends moving in opposite directions (increasing antimicrobial resistance and declining novel antimicrobial development) have precipitated a looming crisis: the nearly complete inability to safely and effectively treat bacterial infections. To avert this, new approaches are needed. One idea is to stimulate host defense pathways to improve the clearance of bacterial infection. Here, we describe five small molecules that promote resistance to infectious bacteria by activating C. elegans’ innate immune pathways. Several are effective against both Gram-positive and Gram-negative pathogens. One of the compounds was mapped to the action of MDT-15/MED15 and NHR-49/HNF4, a pair of transcriptional regulators more generally associated with fatty acid metabolism, potentially highlighting a new link between these biological functions. These studies pave the way for future characterization of the anti-infective activity of the molecules in higher organisms and highlight the compounds’ potential utility for further investigation of immune modulation as a novel therapeutic approach.

## Abstract (precis)

Trends moving in opposite directions (increasing antimicrobial resistance and declining novel antimicrobial development) have precipitated a looming crisis: a nearly complete inability to safely and effectively treat bacterial infections. To avert this, new approaches are needed.

## INTRODUCTION

Pseudomonas aeruginosa is an opportunistic human pathogen that presents a serious problem for patients with weakened immune systems, severe burns, or cystic fibrosis (1, 2). Infection frequently occurs in hospital settings and typically involves multidrug-resistant strains that are insensitive to frontline treatments like β-lactams or aminoglycosides. Problematically, the pathogen is inherently resistant to many classes of antimicrobials and readily acquires new resistance mechanisms via horizontal gene transfer. As a consequence, the number of treatments available continues to ebb.

Unfortunately, the number of pharmaceutical companies pursuing antimicrobial agents, and hence the number of new drug applications for novel antimicrobials, has been dwindling for decades (3). From 1980 to 1990, at least 30 new drug applications were filed for antimicrobials, while the decade from 2000 to 2010 yielded only 7 (4). Alternative therapies to combat the growing threat of antimicrobial resistance are sorely needed.

One potential approach to this problem is to stimulate host immune pathways to promote defense. A more effective defense may minimize, or even prevent, the spread of infection in the body, limiting the damage to the host and allowing the healing process to begin. This also has the side benefit of reducing the pressure placed on the pathogen to evolve resistance, since the drugs target the host instead. The use of immunostimulatory compounds is increasingly common, as well. For example, recombinant cytokines like alpha interferon (IFN-α) and IFN-β are used to modulate the immune response to chronic hepatitis B and hepatitis C viruses, while TLR7 agonists are used in cancer immunotherapy (5–7). Promisingly, both lyophilized bacteria and bacterial lysates have been shown to effectively prevent bacterial infection (8), although these treatments occurred prior to exposure. Immunostimulatory compounds may be a fertile area to search for effective alternative treatments for multidrug-resistant pathogens.

Traditional drug discovery typically involves identification of a promising, druggable target and then screening tens of thousands to millions of compounds to identify those that bind with the highest affinity (9–11). Although this method can be effective, it is beset by several shortcomings. First, the assays are often done *in vitro*, which does not always accurately predict *in vivo* activity. Second, *in vitro* assays are rarely informative about toxicity or bioavailability. Third, despite significant attempts to remove them from screening libraries, *in vitro* screening hits are notoriously plagued by pan-assay interference compounds (PAINS), which are classes of small molecules (e.g., covalent modifiers, chelators, etc.) that appear as false hits in a disproportionate number of drug screens (12, 13). PAINS are often pursued in futile drug development efforts before it becomes clear that their chemistry is unsuitable for biomedical use because of unavoidable off-target activities (12, 13). Fourth, despite the relatively high number of compounds used, these libraries usually still explore a relatively restricted portion of chemical space. Finally, screening conditions very rarely recapitulate host-pathogen interactions. This ignores the potential for either participant to metabolize the compound into a toxic or ineffective metabolite and squanders the opportunity to identify disruptors of these interactions.

New approaches have been developed to address these concerns. Phenotypic and high-content screens, for example, have rapidly gained popularity. These methods use cells, or even whole organisms, as a screening population. As digital storage and computer analysis have become less expensive and more powerful, screening criteria have also become more complex, including measures such as cell or organism viability, ultrastructural details, or even image-based phenotypes. One clear advantage of these methodologies is that host viability can be used as a hit criterion, which rapidly eliminates toxic or biologically unavailable compounds from the pool of hits.

Another advantage of these screens is that they have the potential to simultaneously identify compounds targeting multiple host and pathogen biochemical pathways. If both host and pathogen are present, immune stimulators may be identified as well, since whole organisms can be screened for the activation of desired immune responses with real-time readout of fluorescence or luminescence (14, 15). There has also been a shift in the chemical libraries used for screening from large, complex molecules that very tightly interact with their targets to smaller, more nimble fragments that will have lower affinity but are also less likely to be completely blocked by steric inhibition if they do not have an ideal fit. This shift allows even weak, partial matches to provide some information that can be used for lead development.

Caenorhabditis elegans represents a nearly ideal host for these screens. In addition to its other well-known benefits as a model organism (simple genetic manipulation, large number of progeny, short generation time, tremendous available knowledge about host biology, and its transparent body), it combines a small size (allowing for assay miniaturization and screening in 384-well plates) with differentiated tissues for neurological, digestive, muscular, and reproductive function. Finally, its innate immune system shares many features with mammals, including the p38 mitogen-activated protein kinase (MAPK), β-catenin, and FOXO pathways (16). Despite the evolutionary distance between C. elegans and humans, host-pathogen interactions are surprisingly similar (17).

We previously carried out a high-throughput, high-content, fragment-based phenotypic screen for small molecules capable of extending C. elegans survival during exposure to P. aeruginosa in liquid (14). In the process, ∼70 novel small molecules were identified, some of which possessed antibacterial or anti-virulence properties (14, 18, 19). However, a number of hits had no apparent effect on bacterial growth (suggesting that they are not antimicrobials) and also did not prevent the production or the function of pyoverdine (the most important virulence determinant in the assay used for the screen). Therefore, we hypothesized that at least some of these molecules may improve C. elegans survival by augmenting host defense responses.

In this study, we report the identification of five molecules, here called LK32, LK34, LK35, LK38, and LK56 stimulators of innate immunity in C. elegans. All five promoted host survival during exposure to P. aeruginosa in liquid killing, while LK32, LK34, LK38, and LK56 also restricted host killing in a classical slow-kill assay with P. aeruginosa. LK32, LK34, and LK56 improved resistance to Enterococcus faecalis, and LK34 and LK56 conferred resistance to Staphylococcus aureus as well. All four assays use different virulence determinants, indicating the most likely explanation is increased host immune function. Transcriptional profiling indicated that each compound activated a variety of host stress and innate immune effectors. A genetic mechanism was identified for the function of LK56 in rescue against P. aeruginosa, E. faecalis, and S. aureus in liquid, which uses MDT-15/MED15 and NHR-49/HNF4. Although both of these genes have been implicated in defense in C. elegans, this is the first report of the two of them participating in the same process in innate immunity. We also determined that LK38 depends on the PMK-1/p38 MAPK pathway (and its upstream members NSY-1/MAP3K and SEK-1/MAPKK) in slow killing.

## RESULTS

### Identification of potential immunostimulants.

For the first round of characterization, 69 novel small molecules previously selected on the basis of their ability to improve C. elegans survival during exposure to P. aeruginosa in liquid (14) were tested for the ability to interfere with bacterial growth. MICs were determined for each compound by growing P. aeruginosa strain PA14 in static culture in 384-well plates with serial 2-fold dilutions of compounds. No worms were used in these assays. Next, effective rescue concentrations (EC; defined as the minimum concentration that results in statistically significant survival, compared to dimethyl sulfoxide [DMSO]) (15, 18) were determined for each compound using the standard P. aeruginosa liquid killing assay. Sterile *glp-4(bn2)* worms were used in all liquid-based assays to limit artifacts (i.e., unlaid eggs will hatch and cause matricide).

The ratio of MIC to EC was determined for each of the 69 compounds. We have previously used this as a simple way to identify compounds whose salubrious effects are primarily driven by limiting bacterial growth. For example, since antimicrobials’ mechanism of rescuing C. elegans’ death is contingent on preventing bacterial growth, they generally have MIC/EC ratios close to 1.0. Ciprofloxacin, gentamicin, and polymyxin B, for example, are conventional antimicrobials that kill P. aeruginosa and rescue worms in the liquid killing assay. These drugs have MIC/EC ratios that range from 0.57 to 2.7 (18). In contrast, compounds that prevent pyoverdine biosynthesis or function, such as 5-fluorocytosine, LK11, LK31a, and PQ3c, exhibit MIC/EC ratios that range from 15 to >35 (14, 18, 19). For compounds with an MIC/EC ratio of >10, indicating a nonantimicrobial mechanism, the expression of 115 genes involved in C. elegans host defense pathways was evaluated using a previously designed custom nanoString codeset (18). Based on these data, about 20 small molecules upregulated host defense pathways.

**FIG 1:**
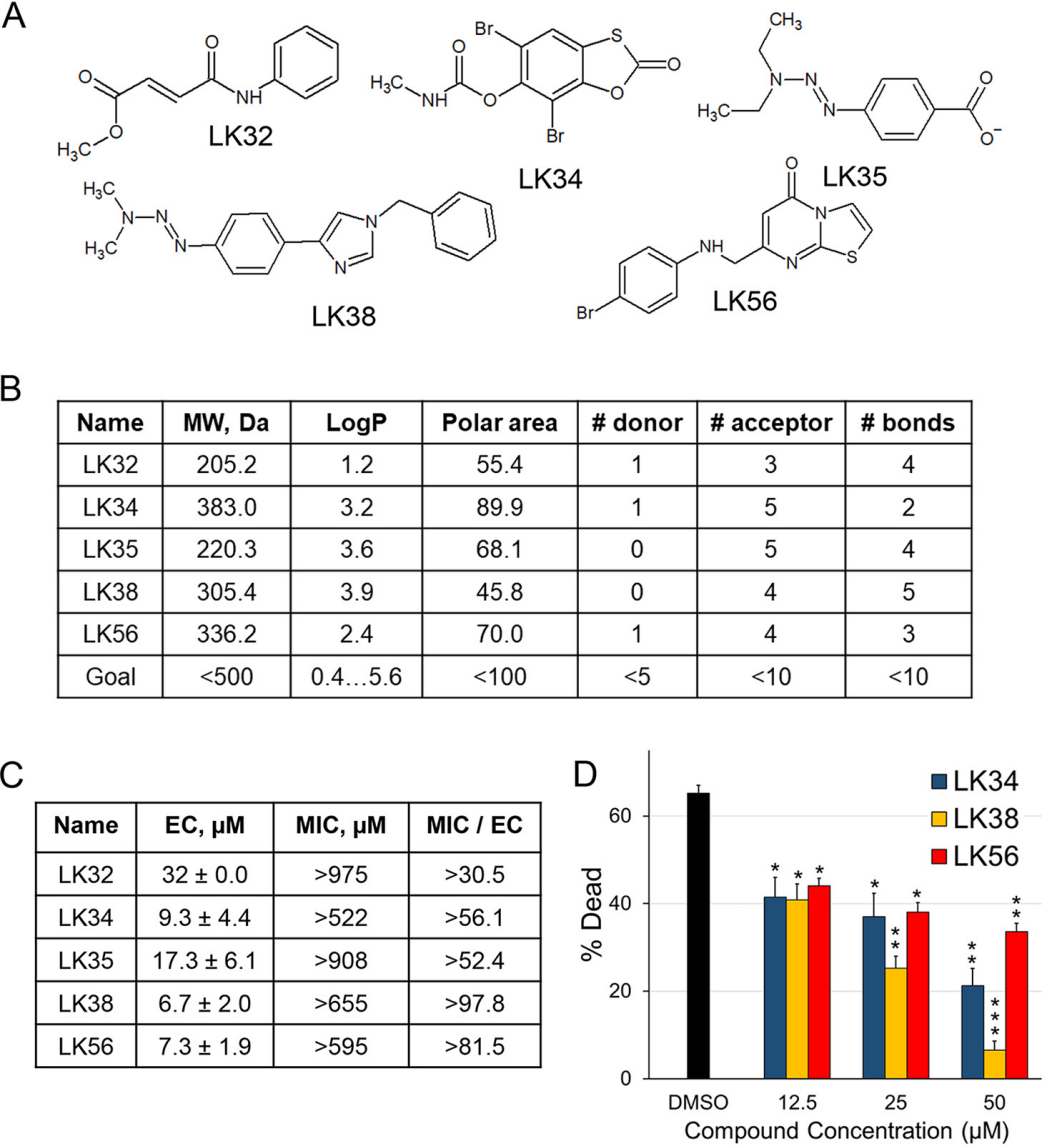
LK molecules rescue C. elegans from P. aeruginosa in liquid killing. (A) Structures for the five LK molecules—LK32, LK34, LK35, LK38, and LK56—are shown. (B) Lipinski values for the five LK molecules, showing their molecular mass, predicted octanol-water partition coefficient, polar surface area, number of hydrogen donors and acceptors, and the number of rotatable bonds. (C) Minimum effective concentrations (EC) and 95% confidence interval, MICs (defined as the minimum concentration to prevent grown in LB media), and their ratio (MIC/EC). The minimum effective concentration was calculated from the lowest concentration that retained statistically significant rescue of *glp-4(bn2)* C. elegans, a temperature-sensitive sterile strain, from exposure to P. aeruginosa PA14. (D) Young adult *glp-4(bn2)* worms were exposed to P. aeruginosa isolate PA2-61 in the liquid killing assay in the presence of LK34, LK38, or LK56. Compounds were added in serial 2-fold dilutions from 50 µM to 12.5 μM. *, *P* < 0.05; **, *P* < 0.01; ***, *P* < 0.001. *P* values were calculated using Student’s *t* test. At least six wells containing 20 worms each per condition per replicate were used for determination of liquid killing. EC values were calculated based on at least three biological replicates. Error bars represent the standard error of the mean.

Based on MIC/EC ratios, upregulation of C. elegans defense responses, and favorable chemical properties, five small molecules were selected for further study: LK32, LK34, LK35, LK38, and LK56 (the structures are shown in Fig. 1A; the results of the nanoString assay are presented in Table S1 in the supplemental material). Analysis of these compounds using Lipinski’s rules, a simple, empirically derived set of principles commonly used to assess oral bioavailability (20), indicated that the compounds had favorable characteristics for being absorbed through the intestinal lining (Fig. 1B). This is often considered a desirable characteristic early in the drug development pipeline. These compounds showed EC values for C. elegans from P. aeruginosa at low- to mid-micromolar concentrations (Fig. 1C). These concentrations were consistent with values normally seen for primary hits from fragment-based screening due to the smaller drug fragments (21). Rescue in liquid killing was dose dependent, as expected, indicating some level of specificity, with the exception of LK32 (see Fig. S1 in the supplemental material). We also tested these strains against a P. aeruginosa isolate from a pediatric cystic fibrosis patient, P. aeruginosa PA2-61, that we previously characterized (22). LK34, LK38, and LK56 demonstrated dose-dependent rescue in this strain as well (Fig. 1D).

10.1128/mSphere.00950-20.1 FIG S1 Dose-dependent activity of LK compounds. Young adult *glp-4(bn2)* worms were exposed to P. aeruginosa strain PA14 in the liquid killing assay in the presence of serial dilutions of LK32 (A), LK34 (B), LK35 (C), LK38 (D), or LK56 (E). Compounds were added in serial 2-fold dilutions from 64 µM to 2 μM. (F) Example bright-field and fluorescence images used to calculate the proportion of dead worms for each well. In all liquid killing assays, a cell-impermeable dye, Sytox Orange, was used to selectively visualize dead worms. Data shown are the mean of at least three biological repeats. *P* values were determined using Student’s *t* test; *, *P* < 0.05; **, *P* < 0.01; ***, *P* < 0.001. Download FIG S1, PDF file, 0.2 MB. Copyright © 2021 Hummell et al. 2021 Hummell et al. This content is distributed under the terms of the Creative Commons Attribution 4.0 International license.

10.1128/mSphere.00950-20.7 TABLE S1 Induction of C. elegans stress response genes by LK molecules. Analysis of the expression of 115 immune- and stress-responsive genes using nanoString technology. Download Table S1, XLSX file, 0.02 MB. Copyright © 2021 Hummell et al. 2021 Hummell et al. This content is distributed under the terms of the Creative Commons Attribution 4.0 International license.

### LK immunostimulants provide resistance to multiple bacterial pathogens.

Since the compounds did not to appear to modulate pathogen growth or disrupt production of pyoverdine (a siderophore made by P. aeruginosa that is a key virulence factor in the liquid killing assay) (14, 18, 23) but were able to activate host defense mechanisms, the most parsimonious explanation was that they were promoting innate immunity. Therefore, the ability of the compounds to ameliorate other infections was tested.

**FIG 2:**
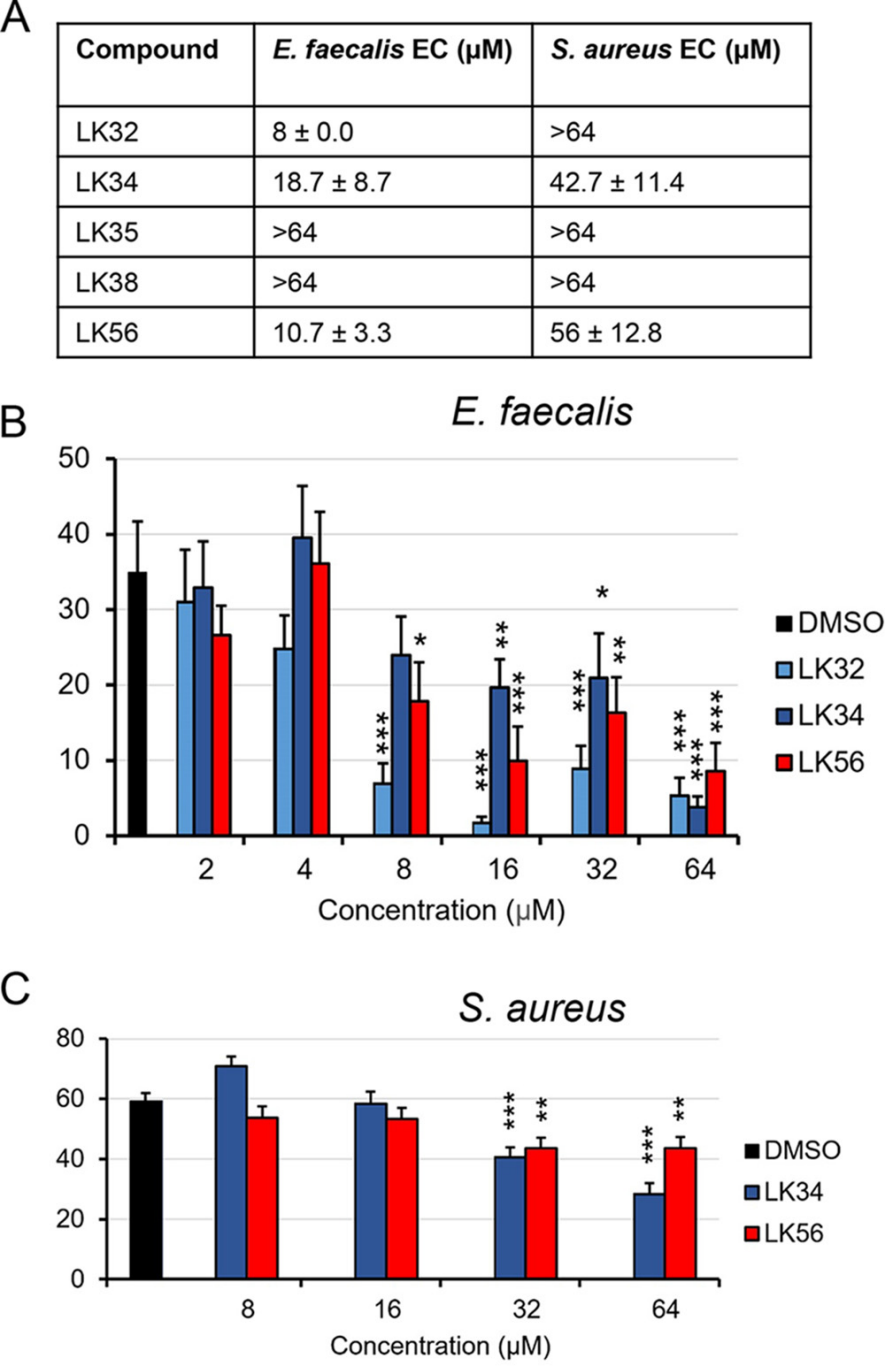
A subset of LK molecules rescue against E. faecalis and S. aureus. (A) Effective rescue concentrations and 95% confidence intervals of LK32, LK34, LK35, LK38, and LK56, as determined by liquid-based infection assays using E. faecalis or S. aureus. Compounds were serially diluted 2-fold, and young adult *glp-4(bn2)* worms were incubated with the pathogen for 80 h (E. faecalis) or 96 h (S. aureus). The concentrations shown are based on the mean value for at least three replicates. (B and C) Percentages of dead C. elegans after exposure to E. faecalis (B) or S. aureus (C). A representative biological replicate is shown. Each condition included at least six wells per replicate, each well contained approximately 20 worms. Three biological replicates were performed. *, *P* < 0.05; **, *P* < 0.01; ***, *P* < 0.001. *P* values were calculated using Student’s *t* test. Error bars represent the standard error of the mean.

Enterococcus faecalis and Staphylococcus aureus are Gram-positive bacterial species frequently responsible for nosocomial infections (24, 25). These bacterial species readily infect C. elegans and liquid-based pathogenesis models have been developed for each (26–28). Sterile, young adult *glp-4(bn2*) worms were incubated with either E. faecalis or S. aureus and various concentrations of the five compounds. Three of the compounds, LK32, LK34, and LK56, showed EC values for E. faecalis (8, 18.7, and 10.7 µM; Fig. 2A) comparable to values for P. aeruginosa (32, 9.3, and 7.3 µM; Fig. 1C) and rescue was dose dependent (Fig. 2B). LK34 and LK56 conferred protection against S. aureus as well but required significantly higher concentrations for rescue than for the other pathogens (42.7 and 56 µM, respectively; Fig. 2C). Importantly, P. aeruginosa, E. faecalis, and S. aureus use very different mechanisms to kill C. elegans (pyoverdine-mediated mitochondrial damage, gelatinase-mediated damage to the colonized intestine, and multi-toxin-mediated intestinal effacement and damage, respectively) (29–33). The ability of these compounds to rescue against multiple pathogens that utilize a diverse set of virulence factors and mechanisms of pathogenesis further supports the idea that at least a portion of these compounds’ activity is mediated by stimulating innate immunity.

### The activity of LK molecules is not mediated by conventional stress response pathways.

The literature linking stress, innate immunity, and aging in C. elegans has long supported a simple model that stress resistance and innate immunity are linked and that the two are inversely correlated with age (34, 35). Although recent, more detailed findings have suggested that it is not quite this simple (36), a strong correlation between stress and pathogen resistance remains. Indeed, it has been clearly demonstrated that stress-inducing compounds can promote pathogen resistance, albeit with some adverse effects on the host. For example, the small synthetic molecule RPW-24 protects C. elegans from P. aeruginosa by activating members of the PMK-1/p38 MAPK pathway, but long-term exposure to the concentration required for rescue shortens life span (16).

**FIG 3:**
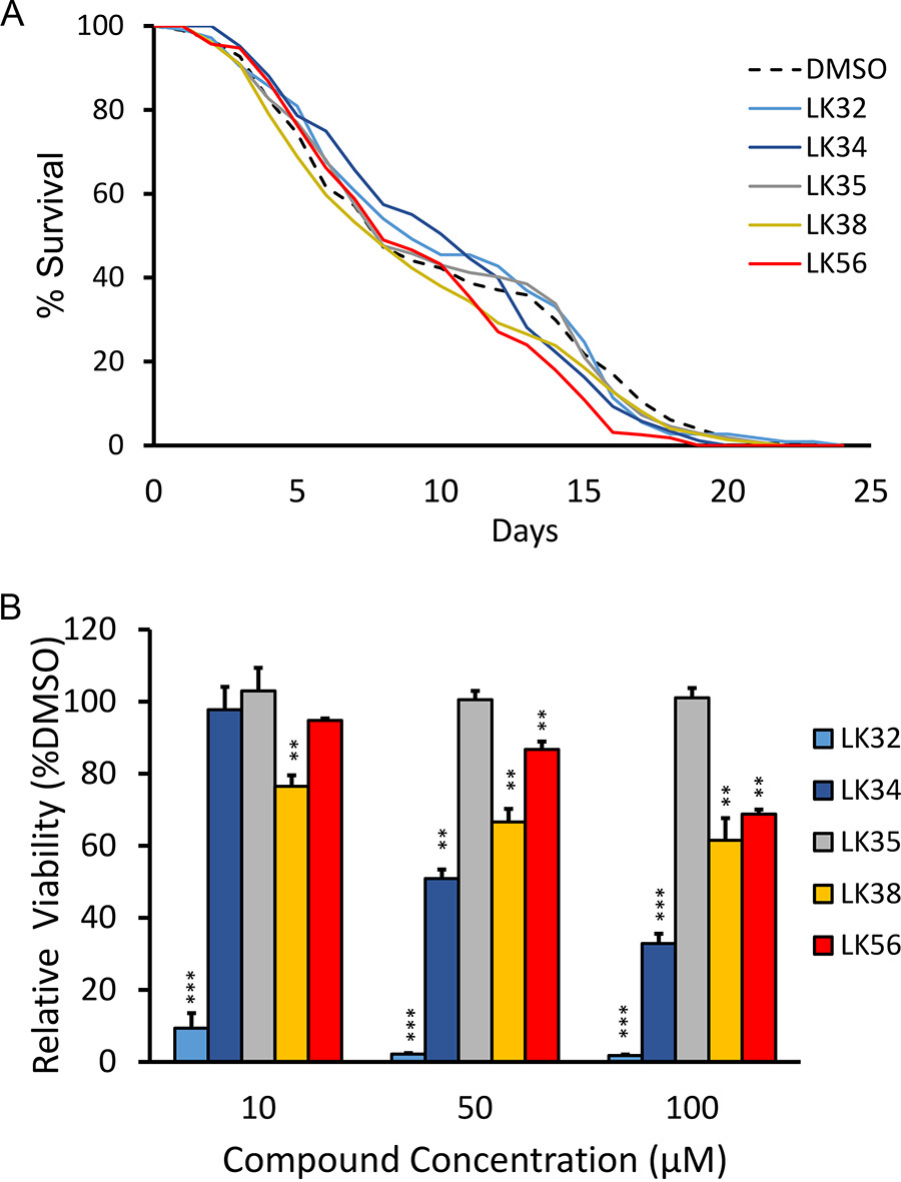
LK molecules exhibit low toxicity in C. elegans and mammalian cells. (A) For each compound, ∼60 *glp-4(bn2)* worms were plated onto each of three NGM plates supplemented with LK32, LK34, LK35, LK38, LK56, or DMSO (50 µM). Worms were scored daily by prodding. Only LK56 showed a statistically significant decrease in life span (*P* < 0.05). Data from a representative biological replicate (one of three total performed) are shown as a Kaplan-Meier plot. (B) 1.5 × 10^4^ RWPE-1 immortalized prostate cells were seeded into each well of a 96-well plate. After 24 h, the medium was replaced with serum-free medium containing LK32, LK34, LK35, LK38, LK56, or DMSO at 10, 50, or 100 µM. Cell viability was determined 24 h later by conventional MTT assays. The viability was normalized to a DMSO solvent control. Six wells were used per condition, and an average of three biological replicates is shown. The *P* value was calculated using either a log-rank test (A; see Table S2 in the supplemental material) or Student’s *t* test (B). *, *P* < 0.05; **, *P* < 0.01; ***, *P* < 0.001. Error bars represent standard error of the mean.

To test the long-term toxicity of the hit compounds, life span assays were carried out by placing sterile, young adult *glp-4(bn2)* worms on NGM (standard nematode growth media, defined in Materials and Methods) seeded with Escherichia coli OP50. LK32, LK34, LK35, LK38, LK56, or DMSO at 50 μM was added to the media during pouring. Sterile worms were used to eliminate the need to transfer worms between plates during life span assays, and we elected to use *glp-4(bn2)* to induce sterility instead of using wild-type worms sterilized with 5-fluoro-2′-deoxyuridine (FUDR) because the interaction of the compounds could generate artifacts during the long course of life span experiments. Of the compounds tested, only LK56 exhibited a slight decrease in life span, and that effect appeared only as the worms reached the end of their life span (Fig. 3A; see Table S2 in the supplemental material for TD_50_ [time to 50% death] and *P* values for individual compounds).

We also tested compound toxicity in RWPE-1 cells, a well-established, noncancerous, immortalized prostate epithelial cell line available in the lab. Cells were seeded in 96-well plates and allowed to attach before media containing various concentrations of one of the compounds (or DMSO as a control) were added. Viability was measured after 24 h later using a standard MTT [3-(4,5-dimethyl-2-thiazolyl)-2,5-diphenyl-2*H*-tetrazolium bromide] assay. Cell death greater than 30% was considered evidence of toxicity (Fig. 3B). In general, human cells showed greater sensitivity to the compounds than C. elegans. This was expected, since the cuticle of C. elegans tends to increase resistance to many substances compared to mammalian cells. However, even the lowest concentration of LK32 was poorly tolerated by the cells. This is consistent with publicly available data in the PubChem database indicating that LK32 was toxic to two human acute lymphoblastic leukemia cell lines (CCRF-CEM and MOLT-4) at concentrations that were close to the measured EC in liquid killing (37).

Although the limited impact of the hits on C. elegans life span suggested that they are mediating their effect through immune stimulation and not by weak toxicity, it remained possible that the compounds were causing subacute levels of specific stress responses that could promote surveillance pathways that also promote pathogen resistance. For this reason, we tested whether LK35, LK38, or LK56 activate a panel of known stress response pathways in the absence of pathogens. LK34 was left out of the following experiments since very little compound remained and it was no longer commercially available. The remaining compound was used for transcriptome profiling (below).

Previous work from our lab has established that the ESRE network plays an important role in the resistance of C. elegans to pyoverdine, the main virulence determinant in liquid killing, and recent evidence has shown that ESRE activation depends on increased reactive oxygen species (ROS) (23, 38, 39). To evaluate ESRE activation, worms carrying an *hsp-16.1p*::GFP reporter (which contains two ESRE motifs and was previously used as an indicator of ESRE activation [39–41]), were exposed to 50 or 100 μM LK35, LK38, LK56, juglone (positive control [38, 42]), or DMSO. Only treatment with LK56 at 100 μM resulted in weak activation of ESRE (see Fig. S2A in the supplemental material). ROS level was assessed based on the fluorescence of dihydroethidium (DHE; a ROS-specific dye) using a COPAS FlowSort for flow vermimetry. Although the positive control (rotenone) showed a significant increase in staining, none of the other compounds exhibited any sign of increased ROS (see Fig. S2B).

10.1128/mSphere.00950-20.2 FIG S2 LK molecules do not appear to perturb cellular proteostasis. Young adult wild-type (A), *hsp-4p*::GFP (B), *hsp-16.1p*::GFP (C), or *rpt-3*::GFP (D) worms were incubated with LK molecules at 50 or 100 µM, DMSO control, or positive controls. For panel A, the worms were treated with compound for 10 h, and then the worms were stained with 4 µM DHE for 1 h before washing and fluorescence measurement. For panels B to D, the worms were treated with compound for 15 h prior to fluorescence measurement. Mean values from at least three biological replicates are shown. *, *P* < 0.05; **, *P* < 0.01; ***, *P* < 0.001 (compared to the solvent control). *P* values were calculated using Student’s *t* test. Error bars represent the standard error of the mean. Download FIG S2, PDF file, 0.2 MB. Copyright © 2021 Hummell et al. 2021 Hummell et al. This content is distributed under the terms of the Creative Commons Attribution 4.0 International license.

In a similar approach, we tested for UPR^ER^ stress using a GFP transcriptional reporter for *hsp-4*, the C. elegans homolog of BiP (see Fig. S2C) and proteasomal stress using an *rpt-3p*::*GFP* reporter (see Fig. S2D). None of the compounds activated these pathways at either concentration. In each case, positive controls confirmed that the reporters were working correctly. These data suggest that the activity of these compounds was unlikely to be triggered by nonspecific stresses.

### Transcriptional analysis of LK immunostimulants indicates shared activities.

To gain additional insight into the effect of LK molecules on C. elegans, young-adult, wild-type worms were treated with each drug at 100 µM for 8 h in the absence of pathogen. RNA was collected, and transcriptome profiling was performed. Gene ontology analysis of upregulated genes identified innate immune responses and lipid storage as statistically significant categories among upregulated and downregulated genes, respectively (see Table S3 in the supplemental material for the list of up- and downregulated genes and Table S4 for Gene Ontology enrichment). Interestingly, alterations in lipid metabolism have been increasingly linked to pathogen response recently (43–45).

10.1128/mSphere.00950-20.8 TABLE S2 TD_50_ quantification. TD_50_ values for the survival and longevity experiments presented in Fig. 3A, Fig. 8, Fig. 9, and Fig. S5. Download Table S2, XLSX file, 0.1 MB. Copyright © 2021 Hummell et al. 2021 Hummell et al. This content is distributed under the terms of the Creative Commons Attribution 4.0 International license.

10.1128/mSphere.00950-20.9 TABLE S3 Lists of differentially expressed genes and their fold changes upon treatment with LK32, LK34, Lk35, LK 38, or LK56. Differentially expressed genes and fold changes after 8 h of treatment with LK individual molecules are shown, in addition to shared targets for LK34, LK34, and LK38. Data were determined using Affymetrix microarrays, as described in Materials and Methods. Download Table S3, XLSX file, 0.07 MB. Copyright © 2021 Hummell et al. 2021 Hummell et al. This content is distributed under the terms of the Creative Commons Attribution 4.0 International license.

10.1128/mSphere.00950-20.10 TABLE S4 Enriched gene ontology categories. Gene ontology enrichment for genes upregulated or downregulated following exposure to LK molecules was determined using DAVID database. Redundant gene ontology categories were removed. Download Table S4, XLSX file, 0.02 MB. Copyright © 2021 Hummell et al. 2021 Hummell et al. This content is distributed under the terms of the Creative Commons Attribution 4.0 International license.

We examined differentially expressed genes to see whether expression changes matched known effectors for well-characterized innate immune pathways (46–51). Each of the molecules shared significant expression patterns with several of the pathways examined (Table 1), indicating (but not proving) that these pathways may be activated.

**TABLE 1:** Statistically significant immune pathways

Molecule	Regulatory control			
	DAF-16 dependent	PMK-1 dependent	SKN-1 dependent	ELT-2 targets
LK32	2.24E–04	1.13E–12	1.60E–12	2.28E−24
LK34	4.63E–18	4.48E–17	9.61E–17	1.49E–12
LK35	5.95E–12	1.08E–11	1.10E–05	1.59E–07
LK38	4.69E–19	2.23E−22	1.23E−14	7.27E−17
LK56	1.18E–09	6.25E–08	3.38E−18	9.29E−18

**FIG 4:**
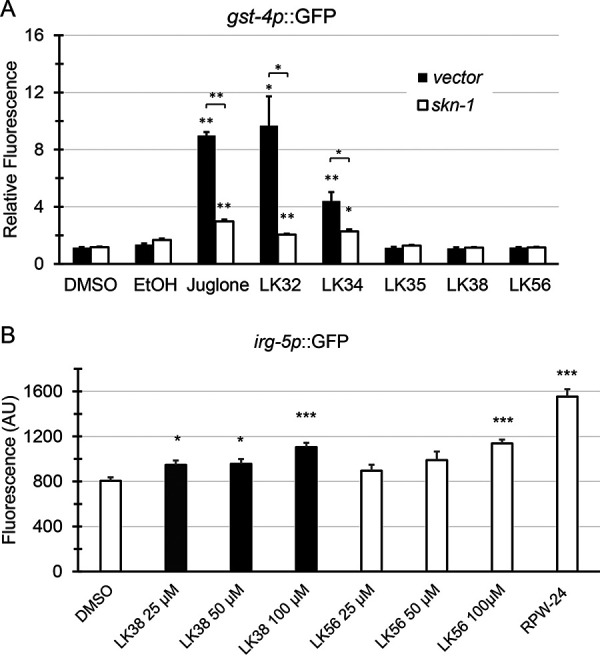
A subset of LK molecules activates reporters for the SKN-1/Nrf2 or PMK-1/p38 MAPK pathways. (A) Worms carrying the *gst-4p*::GFP reporters for SKN-1/Nrf transcriptional activation were reared on vector or *skn-1(RNAi)* and then exposed to LK32, LK34, LK35, LK38, LK56, or juglone (as a positive control) at 100 μM in 1% DMSO. Also present are DMSO and ethanol negative controls for LK compounds and juglone, respectively. The fluorescence intensity was normalized to that of DMSO. (B) Worms carrying an *irg-5p*::GFP reporter for PMK-1/p38 MAPK activity were incubated at 25, 50, or 100 μM in 1% DMSO. Positive and negative controls consisted of 100 μM RPW-24 in 1% DMSO and 1% DMSO alone. Fluorescence was measured at 24 h. For both panels, ∼50 worms were used per well, and at least three wells were used per condition for each biological replicate. Mean values from at least three biological replicates are shown. *, *P* < 0.05; **, *P* < 0.01; ***, *P* < 0.001 compared to the solvent control. *P* values were calculated using Student’s *t* test. Error bars represent the standard error of the mean.

To test activation of these pathways, worms carrying GFP-based reporters for the SKN-1/Nrf, PMK-1/p38 MAPK, and DAF-16/FOXO pathways (*gst-4p*::GFP, *irg-5p*::GFP, and DAF-16::GFP, respectively) were exposed to each compound in S Basal medium in the absence of the pathogen. LK32 and LK34 induced *gst-4p*::GFP in an SKN-1-dependent manner, suggesting *bona fide* activation of the SKN-1/Nrf pathway (Fig. 4A). LK38 and LK56 each triggered a modest increase in *irg-5p*::GFP fluorescence, indicating that they activate the PMK-1/p38 MAPK pathway. Notably, LK38 was able to activate the pathway at concentrations close to the EC, while LK56 only exhibited PMK-1/p38 MAPK activation at higher concentrations (Fig. 4B). The DAF-16::GFP reporter partially translocates from the cytoplasm to the nucleus due to immersion of the worms (see DMSO control), but none of the LK compounds increased this shift (see Fig. S3 in the supplemental material).

10.1128/mSphere.00950-20.3 FIG S3 LK molecules do not stimulate DAF-16/FOXO nuclear localization. (A and B) Examples of worm scored as exhibiting nuclear (A) or cytoplasmic (B) localizations for DAF-16/FOXO::GFP. Worms were scored as either nuclear localization (+) or cytoplasmic localization (–) based on whether at least five distinct GFP fluorescent nuclei were observed. (C) Worms carrying a DAF-16::GFP transcriptional fusion reporter were exposed to LK32, LK34, LK35, LK38, LK56, or juglone (positive control) at 100 µM for 8 h. GFP localization was then scored. At least three biological replicates were performed, with each replicate including ∼30 worms. Error bars represent standard error of the mean. *, *P* < 0.05. *P* values were calculated using Student’s *t* test. Download FIG S3, PDF file, 0.1 MB. Copyright © 2021 Hummell et al. 2021 Hummell et al. This content is distributed under the terms of the Creative Commons Attribution 4.0 International license.

**FIG 5:**
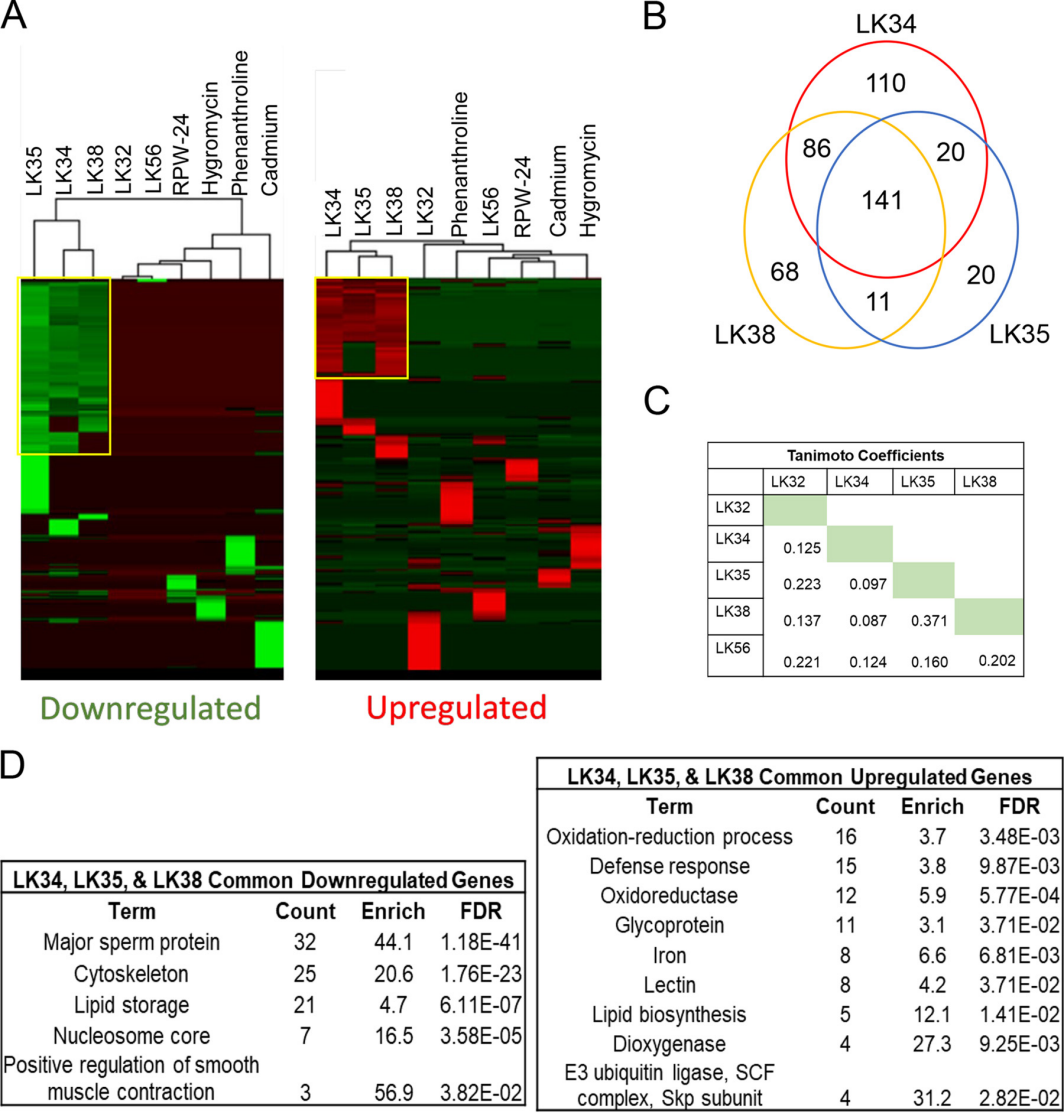
LK34, LK35 and LK38 share common pathways responsible for defense response. (A) Heat map of upregulated and downregulated genes after normalization to average fold change across all conditions. (B) Venn diagram of genes upregulated by LK34, LK35, or LK38. (C) Pairwise compound Tanimoto coefficients calculated using OpenBabel (see Materials and Methods). (D) Gene Ontology terms for upregulated and downregulated genes from the LK34, LK35, and LK38 common set.

Although data on the significance of overlaps between differentially regulated genes (as shown in Table 1) can sometimes provide direction for further investigation, merely comparing lists is often not very informative. Therefore, we also considered the magnitude of the differences in expression. Differentially expressed genes were clustered based on the degree of change after treatment with one of the five immunostimulants or with a panel of compounds known to affect C. elegans, including hygromycin (a translational inhibitor causing proteotoxic stress), RPW-24 (a synthetic small molecule that activates members of the PMK-1/p38 MAPK pathway), and phenanthroline (a metal chelator that mimics pyoverdine exposure and activates mitochondrial surveillance) (15, 30, 39, 52). Interestingly, LK34, LK35, and LK38 clustered together and showed similar gene expression profiles, as reflected by the clustograms and numbers of shared genes and gene categories (Fig. 5A, B, and D). The most obvious explanation for this is that the molecules have a shared chemical structure. We used an implementation of the FP2 algorithm (based on linear segments of the small molecule that include up to seven atoms) in the OpenBabel software package (http://openbabel.org) to evaluate the compounds’ Tanimoto coefficients. These values represent a statistical measure of the chemicals’ similarity. However, the highest pairwise Tanimoto coefficient was only 0.37 (Fig. 5C). This is lower than the most common cutoffs considered to indicate a close structural relationship between the molecules (0.55) or that they share an activity and a target (0.85) (53). However, the transcriptional overlap suggests that the compounds are likely to activate the same pathways.

### Disrupting a single genetic pathway generally did not compromise the rescuing activity of most LK drugs.

**FIG 6:**
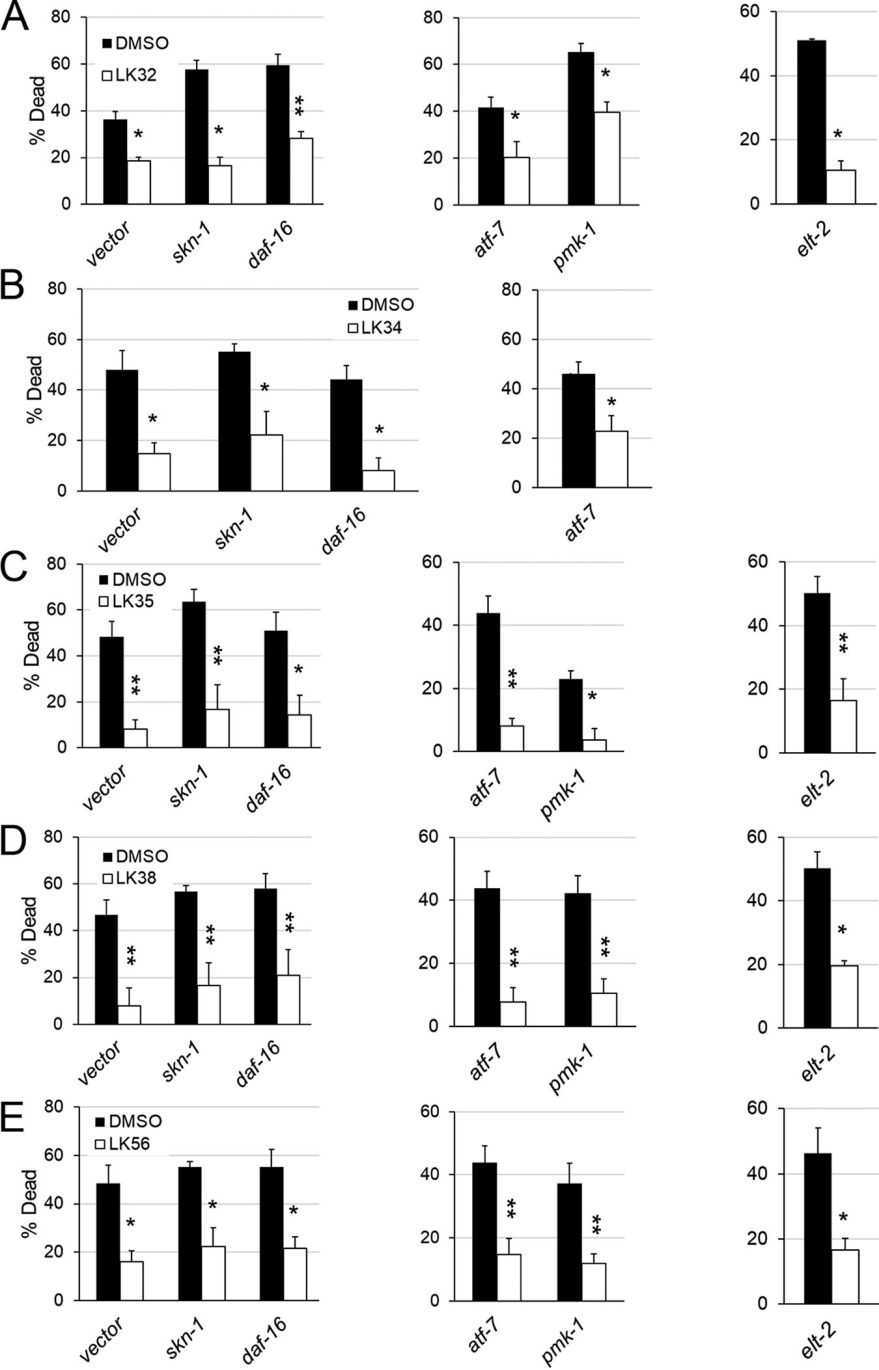
Rescue in liquid killing does not depend upon single canonical innate immune pathway. (A to E) Liquid killing of *glp-4(bn2)* worms reared on RNAi-targeting *skn-1/Nrf2*, *atf-7/ATF5*, *daf-16/FOXO*, *pmk-1/p38 MAPK*, *elt-2/GATA*, or vector RNAi were treated with DMSO or DMSO containing LK32 (A), LK34 (B), LK35 (C), LK38 (D), or LK56 (E). RNAi conditions measured at different time points are represented by different graphs within the same panel. The concentration was either 32 µM (LK38 and LK56) or 64 μM (LK32, LK34, and LK35), depending on which most consistently rescued. At least six wells, containing 20 worms each, were used per biological replicate to determine survival for each condition, and averages from at least three biological replicates are shown. *, *P* < 0.05; **, *P* < 0.01; ***, *P* < 0.001. *P* values were calculated using Student’s *t* test. Error bars represent standard error of the mean.

Based on the presence of effectors for well-known innate immune pathways in the transcriptional profiles of C. elegans exposed to LK molecules, we predicted that the compounds were acting through one or more of these pathways, despite the low levels of reporter activation. To test this, RNAi was used to knock down *daf-16/FOXO*, *elt-2/GATA*, *pmk-1/p38 MAPK*, *atf-7*/*ATF7*, or *skn-1/Nrf2*. Sterile, young adult *glp-4(bn2)* worms were then exposed to P. aeruginosa under liquid killing conditions in the presence of either DMSO or one of the immunostimulatory compounds. Surprisingly, none of the RNA interference (RNAi) conditions tested completely eliminated the ability of the LK compounds to rescue C. elegans (Fig. 6).

It is worth noting that depletion of several of these transcripts via RNAi is known to alter the timing of P. aeruginosa-mediated liquid killing. For example, *daf-16/FOXO(RNAi)* compromises survival in liquid media, even in the absence of P. aeruginosa, as DAF-16 provides some resistance to the stress of immersion. This is consistent with our observations that worms incubated in liquid exhibit increased levels of constitutive nuclear translocation of DAF-16/FOXO (30). Consequently, targeting *daf-16/FOXO* with RNAi nonspecifically shortens worm survival in liquid killing.

As an alternative approach, we developed a panel of phosphatases, kinases, transcription factors, and the three genes most upregulated by LK34, LK35, and LK38. RNAi was used to target each of these genes and then worms were exposed to P. aeruginosa in liquid killing conditions in the presence of DMSO, LK34, LK35, or LK38. As with the previous assays, rescue was unchanged (see Fig. S4A in the supplemental material).

10.1128/mSphere.00950-20.4 FIG S4 RNAi does not abolish compound activity. (A) Sterile, young adult *glp-4(bn2)* worms were reared on RNAi targeting *irid-28*, *F38B2.4*, *T24B8.5*, *mex-5*, *Y105C5B.15*, *comt-4*, or empty vector and then exposed to P. aeruginosa in the liquid killing assay. (B) Sterile, young adult *glp-4(bn2)* worms were reared on RNAi targeting *met-2*, *osm-8*, *rde-4*, *oga-1*, *daf-2*, *lin-35*, *glp-1*, *dpy-10*, or *dpy-9* and then exposed to P. aeruginosa in the liquid killing assay. These genes were chosen using WormEXP with the criterion that gene expression after mutation would be similar (in terms of upregulation and downregulation) to exposure to LK56. Endpoints were collected at different times (to allow DMSO-treated worms to be approximately 30 to 50% dead). Assays collected at different time points are shown on different graphs. Data show the means from three biological replicates. For each experiment, three biological replicates were performed, with each replicate comprised of six wells with 20 worms/well. Error bars represent standard error of the mean. *, *P* < 0.05; **, *P* < 0.01; ***, *P* < 0.001. *P* values were calculated using Student’s *t* test. Download FIG S4, PDF file, 0.2 MB. Copyright © 2021 Hummell et al. 2021 Hummell et al. This content is distributed under the terms of the Creative Commons Attribution 4.0 International license.

### LK56 requires MDT-15/MED15 and NHR-49/HNF4 for activity.

**FIG 7:**
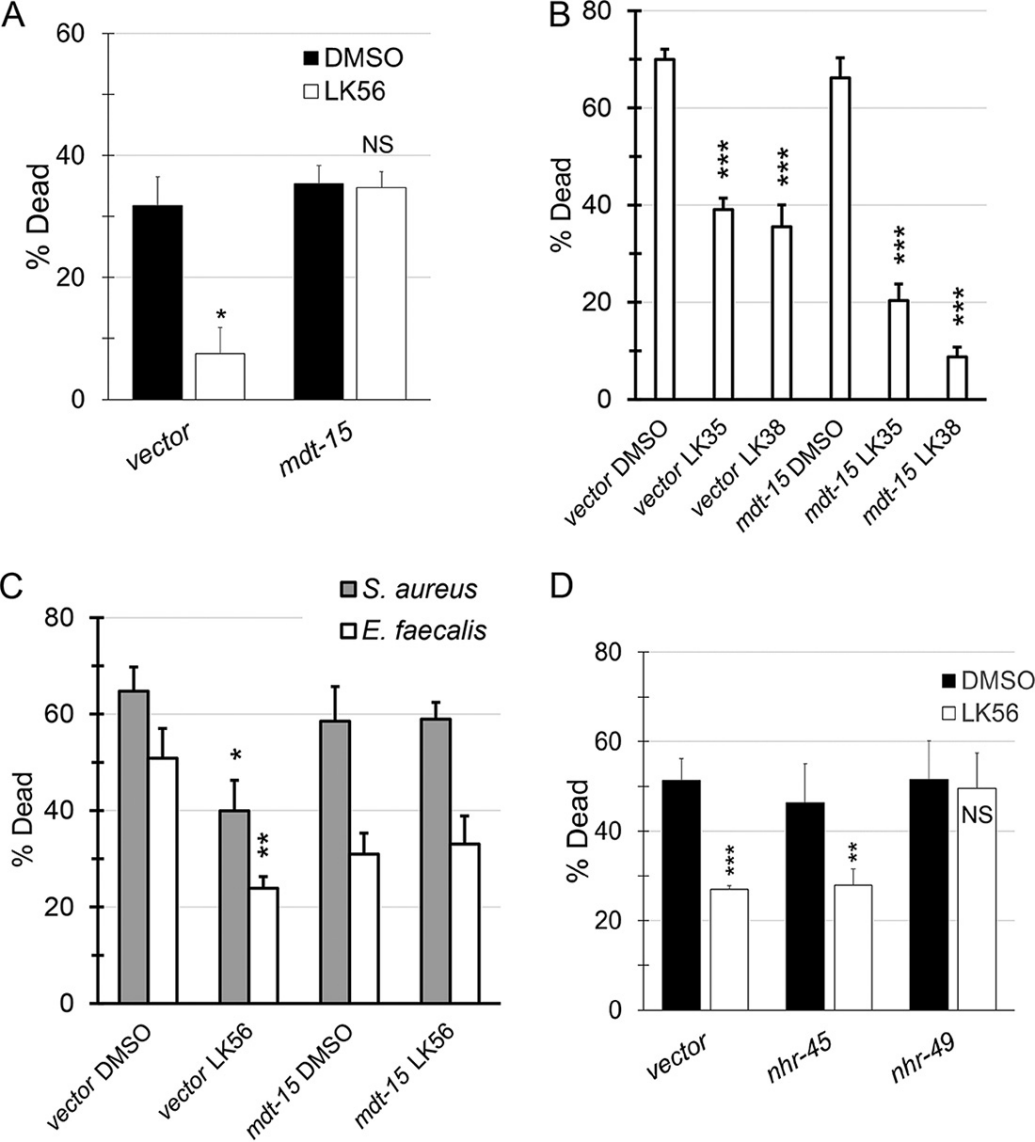
Rescue from pathogens by LK56 requires MDT-125/Med15. (A and B) Young adult *glp-4(bn2)* worms reared on vector or *mdt-15(RNAi)* were incubated with P. aeruginosa strain PA14 in the liquid killing assay with or without LK56 at 32 µM. (B) Young adult *glp-4(bn2)* worms reared on vector or *mdt-15(RNAi)* were exposed to P. aeruginosa PA14 in the liquid killing assay in the presence of LK35, LK38, or DMSO. (C) Young adult *glp-4(bn2)* worms reared on vector or *mdt-15(RNAi)* and then exposed to E. faecalis or S. aureus in liquid-based assays in the presence or absence of LK56 at 32 µM. (D) Young adult *glp-4(bn2)* worms reared on vector, *nhr-49(RNAi)*, or *nhr-45(RNAi)* were incubated with P. aeruginosa PA14 in the liquid killing assay with or without LK56 at 32 µM. Each replicate included at least six wells, with approximately 20 worms per well. The data shown are mean values from a representative replicate. *, *P* < 0.05; **, *P* < 0.01; ***, *P* < 0.001. *P* values were calculated using Student’s *t* test. Error bars represent the standard error of the mean. At least three biological replicates were performed.

Due to its strong rescue and unique transcriptional profile, we also created a panel of genes to test for LK56. We used WormEXP to identify candidate genes whose disruption resulted in patterns of differential gene expression that matched worms treated with LK56. Of the nine genes initially tested (*mdt-15*/MED15, *met-2*/SETDB, *osm-8*, *rde-4*/TARBP2, *oga-1*/OGA, *daf-2*/IGFR, *lin-35*/Rb, *glp-1*/NOTCH, *dpy-9*/COL6, and *dpy-10*/COL6), only *mdt-15/*MED15 disruption was able to completely abolish LK56-dependent rescue (Fig. 7A; see Fig. S4B in the supplemental material). This effect was specific, since *mdt-15(RNAi)* had no effect on the ability of LK35 or LK38 to rescue in this assay (Fig. 7B). Importantly, MDT-15 was also required for LK56-mediated rescue in E. faecalis and S. aureus pathogenesis assays (Fig. 7C).

MDT-15/MED15 is a subunit of the Mediator complex, which is required for gene transcription in all eukaryotes. Unlike other subunits of the complex, MDT-15 specifically regulates a subset of genes. It is best known for its role in regulating fatty acid biosynthesis (54), but it also has been shown to play roles in the response to fasting, heavy metal detoxification, and xenobiotic metabolism and in maintaining mitochondrial homeostasis (55–57).

MDT-15 partners with at least two different nuclear hormone receptors, NHR-45 and NHR-49, to regulate gene expression. Therefore, we also tested whether RNAi targeting these genes would affect LK56-mediated rescue. *nhr-49(RNAi)*, but not *nhr-45(RNAi)*, abolished compound rescue, suggesting that NHR-49/HNF4, like MDT-15/MED15, is required for LK56 activity (Fig. 7D).

Interestingly, MDT-15/MED15 and NHR-49/HNF4 have been independently linked with innate immune defense in C. elegans (58–60), but this is the first time that they have been linked to the same process. An interesting possibility is that fatty acid metabolism, a function commonly associated with both genes (61, 62), underlies LK56 rescue. We would predict that this is independent of the ability of NHR-49 and MDT-15 to activate fatty acid metabolism in response to oxidative stress, however, since several pieces of data suggest that oxidative stress is not particularly prominent during LK56 exposure. First, DHE staining was not increased, suggesting that superoxide and peroxide production were not dramatically increased. Furthermore, a *gst-4p*::GFP reporter was not activated by the compound and our data indicate that SKN-1 is dispensable for LK56-mediated rescue. The possibility that LK56 may tie fatty acid metabolism to innate immunity is tantalizing, but needs further investigation.

### LK38 requires PMK-1/p38 MAPK to protect worms from slow killing.

**FIG 8:**
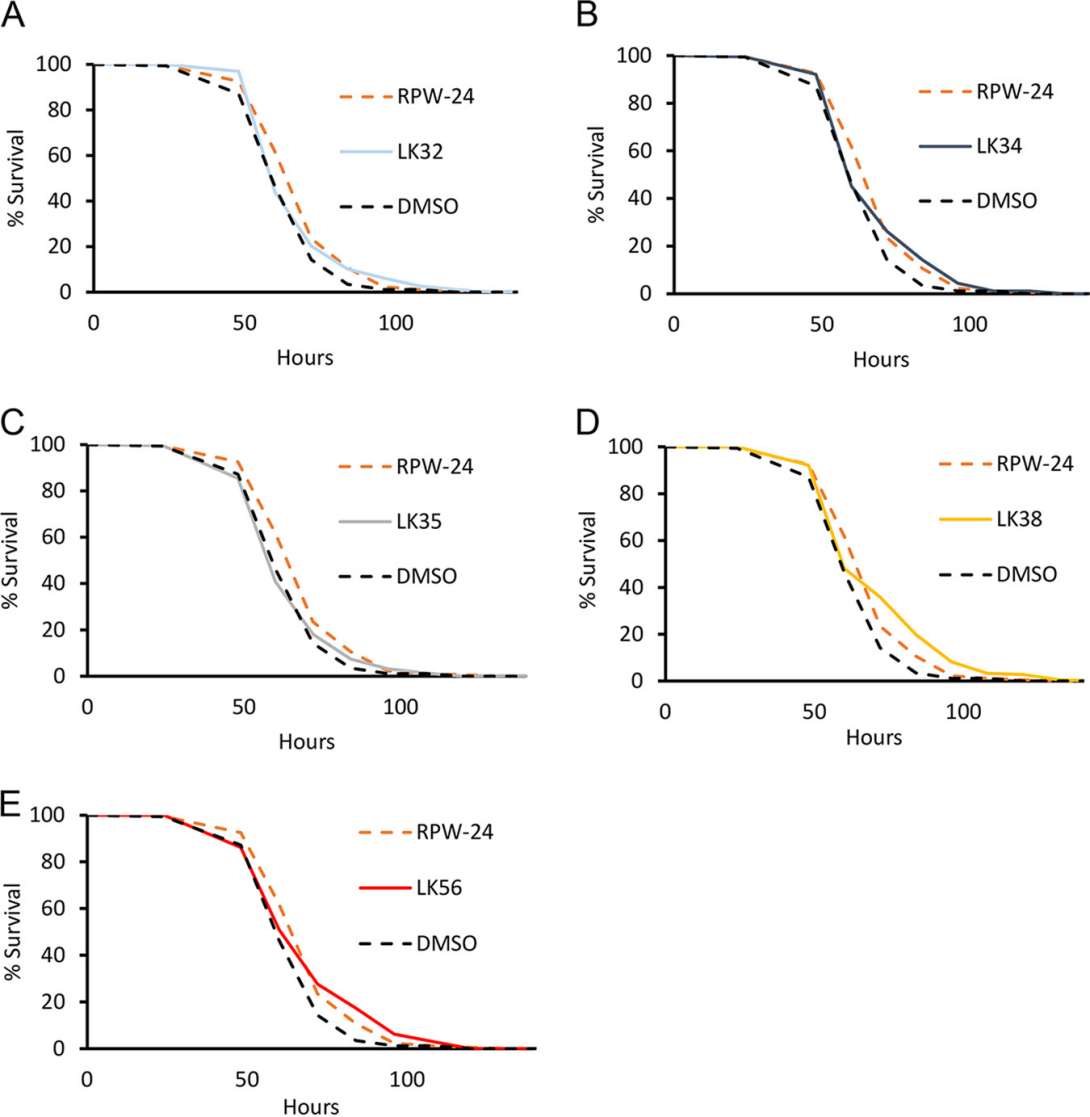
Four LK molecules extend C. elegans life span in the slow-killing assay. Slow-kill assays were performed with young adult, wild-type C. elegans using SK media plates containing LK32 (A), LK34 (B), LK35 (C), LK38 (D), or LK56 (E) or RPW-24 (as a positive control) at 50 µM. The data shown are from a representative replicate. Each of the three biological replicates was comprised of three plates per condition, with each plate containing ∼60 worms. Statistical significance was calculated using a log-rank test. Worms that left the surface of the plate were excluded from analysis (see Table S2 in the supplemental material for TD_50_ and exact *P* values). For DMSO versus compound, *P* < 0.05 for LK32 and LK34, *P* < 0.01 for LK56, *P* < 0.001 for LK38 and RPW-24, and *P* > 0.05 (not significant) for LK35.

**FIG 9:**
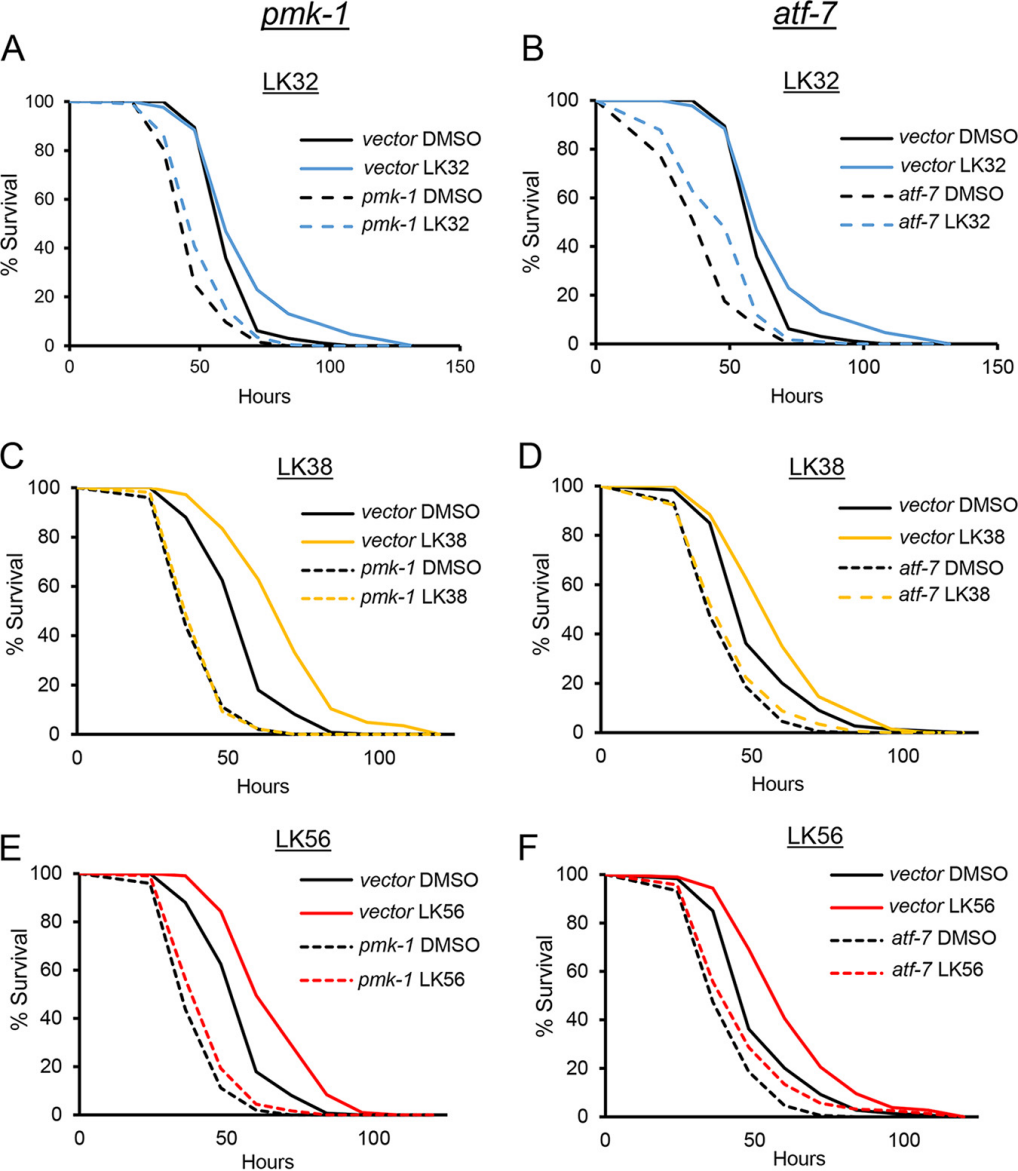
Rescue in slow killing by a subset of LK compounds depends upon the PMK-1/p38 MAPK pathway. Wild-type L4 worms were reared on RNAi targeting *pmk-1*/p38 MAPK, *atf-7/ATF-7*, or empty vector and placed onto slow-killing plates containing DMSO or LK32 (A and B), LK38 (C and D), or LK56 (E and F) at 50 µM. Starting at 24 h, worms were scored every 12 h for survival until all worms perished. Each of the three biological replicates was comprised of three plates, each plate contained approximately 50 to 70 worms. Statistical significance was calculated using log-rank test (see Table S2 in the supplemental material for TD_50_ and exact *P* values). For vector RNAi, DMSO versus compound, *P* < 0.001 for LK38, LK56, and LK32. For *pmk-1/p38 MAPK(RNAi)*, DMSO versus compound, *P* < 0.01 for LK32, *P* < 0.05 for LK56, and *P* > 0.05 (not significant) for LK38. For *atf-7*/*ATF-7*(*RNAi*), DMSO versus compound, *P* < 0.001 for LK32, *P* < 0.01 for LK56, and *P* > 0.05 (not significant) for LK38.

The finding that each of the compounds activated PMK-1/p38 MAPK targets was of interest, as this pathway is associated with enhanced immunity against multiple bacterial pathogens in agar-based C. elegans assays. Therefore, we tested whether these compounds could promote survival in an agar-based, high-colonization pathogenesis model known as slow killing. Loss of PMK-1 activity substantially compromises survival in the slow-killing assay (16, 47). Wild-type, young adult worms were exposed to P. aeruginosa strain PA14 on agar plates impregnated with each of the five compounds. Four of the five compounds (LK32, LK34, LK38, and LK56) improved survival (Fig. 8; see also Table S2 in the supplemental material for the TD_50_ and overall statistical significance). To see whether this depended upon the PMK-1 pathway, LK32, LK38, and LK56 were retested in worms where RNAi was used to knock down *pmk-1*/*MAPK* or *atf-7/ATF7* (a key transcription factor whose activity is modulated by PMK-1 [63, 64]) (Fig. 9; see Table S2 in the supplemental material for TD_50_ and overall statistical significance). As with liquid killing, LK32 and LK56 retained at least partial ability to rescue in spite of disruption of the PMK-1 MAPK pathway via RNAi.

In contrast, LK38 was completely dependent upon the PMK-1/MAPK pathways. This was further confirmed using loss-of-function mutations of *nsy-1/MAP3K(ag3)* and *sek-1/MAP2(km4)*, two MAPK pathway members that are upstream of PMK-1/p38 MAPK (65) (see Fig. S5 in the supplemental material for survival curves and Table S2 for TD_50_ and overall statistical significance). These results suggest that the target of LK38 is upstream or parallel to NSY-1/MAP3K.

## DISCUSSION

### Phenotype-based, host-pathogen screens facilitate the identification of immunostimulatory compounds.

Known innate immune stimulators generally fall into two groups. The first is naturally occurring substances like vaccine adjuvants and agonists of pattern receptors of the innate immune system (TLRs, NLRs, etc.). The second, much smaller, group is composed of synthetic small molecule stimulants like pidotimod, a compound that induces dendritic cell maturation and stimulates the release of proinflammatory cytokines, polarizing CD4^+^ T cells toward a Th1 cell fate (66). Identification of the latter group of compounds is difficult using conventional drug screening methods. However, whole organism phenotypic screening, an approach pioneered by the Ausubel lab using C. elegans and medically relevant bacteria (67, 68), has tremendous promise for identifying these types of compounds.

Using a well-developed C. elegans*-*P. aeruginosa pathosystem, we have previously carried out a moderately sized, phenotype-based screen of several fragment-based small molecule libraries. Hits from this screen appeared to fall into several broad categories, including conventional antimicrobials (i.e., those that prevent bacterial growth) (14, 69), drugs that interfere with bacterial factors required for virulence (e.g., by preventing the production or function of pyoverdine) (18, 19), and those that appear to stimulate host immunity. Here, we characterized five molecules—LK32, LK34, LK35, LK38, and LK56—that fall into this last category.

Several lines of evidence supported this claim, including their ability to mediate rescue against multiple pathogens or against P. aeruginosa in pathogenesis assays with very different, nonoverlapping virulence determinants. The observations that the LK compounds also generally activated multiple innate immune pathways and had high MIC/EC ratios also indicated that at least part of their effects arise from immunostimulatory activities.

### Identification of compound targets from phenotype-based assays is a complex task.

Despite considerable effort, we were only able to conclusively identify the genetic mechanism for LK56 in liquid killing and for LK38 in slow killing. An analogy can be made between target-based and phenotype-based drug screens and reverse and forward genetic screens. While target-based screens and reverse genetics have the advantage of starting with a known target, phenotype-based and forward genetic screens provide the ability to identify hits that have previously unknown roles in the biological process of interest, but at the cost of having targets that are more difficult to identify. In the case of drug screens, this allows all of the hosts’ immune factors to be screened simultaneously. Considering the interplay of the innate immune system (and, in more complex eukaryotes, adaptive immunity as well), it should not be surprising that the compounds discovered this way may have pleiotropic effects.

While attempting to identify potential defense pathways being activated by these immunostimulants, we noted that genes regulated by SKN-1/Nrf, PMK-1/p38 MAPK, and DAF-16/FOXO were statistically overrepresented in the transcriptional profile of each of the compounds. Despite this, transcriptional reporters for these transcription factors (*gst-4p*::GFP, *irg-5p*::GFP, and DAF-16::GFP) were generally activated weakly, if at all. It is difficult to unambiguously explain this discrepancy, but three important possibilities cannot be ruled out.

First, statistical analyses analyze groups of genes, while reporters are typically a single target. Transcription factors rarely operate in a vacuum, and it is common for genes to be under the simultaneous control of more than one such regulator, meaning that several may be required for gene expression. For the same reason, it is common for transcription factors to have subsets of targets, all of which may not be activated by a single stimulus. Consequently, a statistical analysis may identify pathway activation that a reporter test might miss.

It is also worth noting that activation of any of these pathways may be irrelevant (or even counterproductive) to compound-mediated rescue. An extreme example of this is the statistical overrepresentation of PMK-1 targets in the transcriptional profiles of all five compounds. Two of the compounds, LK38 and LK56, even activate *irg-5p*::GFP, a *bona fide* PMK-1 reporter (70). Although this is helpful in some infection contexts (e.g., P. aeruginosa infection on solid media), PMK-1/MAPK activity is actually deleterious for survival in liquid killing, as we have previously established (30). In this particular case, the likeliest explanation is that the beneficial outcomes from compound exposure outweigh the negative effects of activating the PMK-1/MAPK pathway. This contrasts directly with the agar-based slow-killing assay, where LK38-mediated improvement requires this pathway.

Counterintuitively, capturing the messy interplay among biochemical pathways is an advantage, rather than a drawback, of whole organism screening. Medical treatment takes place in a similarly complex milieu, and capturing this complexity early in the process, while sometimes confusing, can also limit investment in hits that perform well in simpler assays but fail in more realistic tests. Although survival is useful as an assay readout in many respects, its binary state can limit its usefulness. Ultimately, the development of more nuanced assay outputs will substantially improve the utility of phenotype-based assays.

### LK immunostimulants likely have other functions.

Although it seems likely that LK32, LK34, LK35, LK38, and LK56 have immunostimulatory activities, it seems clear that they have other activity as well. For example, LK32 showed substantial toxicity against mammalian cells, a finding that has also been reported previously (37). CCRF-CEM and MOLT-4 cell lines, both of which are derived from patients with acute lymphoblastic leukemia, exhibited an LD_50_ of ∼40 μM, which is consistent with what we observed for RWPE-1 prostate cells. In C. elegans, LK32 activated the PMK-1/p38 MAPK, SKN-1/Nrf, and ELT-2/GATA immune pathways, but in the absence of more information, it is difficult to understand why activation of any of these pathways would be toxic. The likeliest explanation is that LK32 has broad-spectrum toxicity and that this activates detoxification functions in C. elegans; it may be also be toxic to bacteria.

LK34 also showed some toxicity in mammalian cells, although C. elegans appeared to be unaffected. LK34 is a member of the 1,3-benzoxathiol-2-one class of compounds, which have been linked to a variety of functions, including antibacterial, antimycotic, antioxidant, antitumor, and anti-inflammatory activities (71–74), and is related to the anti-acne medicine tioxolone. This outcome is also consistent with other reports of LK34 being a potent inhibitor of the GroEL and GroES families of bacterial chaperones (75, 76). Intriguingly, LK34 may also retain some activity against mitochondrial chaperones, which may explain its ability to activate stress response and innate immune pathways in C. elegans. While screening this family of compounds, Johnson and colleagues noted that related compounds were more effective against Gram-positive pathogens (particularly S. aureus) than Gram-negative pathogens, which we also observed in comparing its effect on S. aureus and E. faecalis *versus* P. aeruginosa. Likely its effect in our assay was a combination of modifying bacterial growth (which would reduce pathogenesis) and stimulation of stress and innate immune function, probably through its effect on mitochondria.

Despite their relatively low Tanimoto coefficient (0.371), LK35 and LK38 are apparently related compounds, each sharing an *N*,*N*-dialkylated phenyl triazene substructure (see Fig. S6 in the supplemental material). This is a subgroup of a larger class, the *N*,*N*-dialkylated triazenes, which are well known for their antitumor effects. This appears to be mediated through host compound metabolism, which results in the transfer of a methyl group to the O^6^ position of guanine (O^6^MeG), which disrupts normal base pairing and introduces G:C to A:T transitions (77). This effect has been best studied for the clinically relevant compound dacarbazine, which is used for treatment of recurrent melanoma (78). Efforts to improve the chemistry of dacarbazine led to the synthesis of 1-*p*-carboxy-3,3-dimethylphenyltriazene, also known as CB10-277. This compound shared the activity of dacarbazine and showed promising results in early testing (78). Interestingly, CB10-277 is nearly identical to LK35, with the sole difference being that the triazine in LK35 is diethylated instead of dimethylated.

10.1128/mSphere.00950-20.5 FIG S5 NSY-1 and SEK-1, upstream members of the PMK-1/p38 MAPK pathway, are required for LK38-mediated rescue. Young adult wild-type, *nsy-1/MAP3K15(ag3)*, or *sek-1/MAP2K6(km-4)* mutants were exposed to P. aeruginosa in the slow-killing assay in the presence of DMSO or LK38. Data for each condition at each time point represent the average value for three plates from one representative replicate (of three replicates total). Each plate contained ∼60 worms. *P* values were calculated using a log-rank test (see Table S2 in the supplemental material for the TD_50_ and exact *P* values). For wild-type DMSO versus LK38 *P* < 0.001. No statistically significant difference was measured between LK38 and DMSO for either mutant. Download FIG S5, PDF file, 0.1 MB. Copyright © 2021 Hummell et al. 2021 Hummell et al. This content is distributed under the terms of the Creative Commons Attribution 4.0 International license.

10.1128/mSphere.00950-20.6 FIG S6 Phenyl triazine compounds located in the high-throughput screen. Chemical structures are shown for LK34, LK35, LK36, LK37, LK38, and LK39. The phenyl triazene moiety is indicated in each compound. The nitrogen atom bearing the alkyl groups linked to putative guanine methylation is shown at left for each compound. Download FIG S6, PDF file, 0.10 MB. Copyright © 2021 Hummell et al. 2021 Hummell et al. This content is distributed under the terms of the Creative Commons Attribution 4.0 International license.

It is worth noting that LK35 and LK38 were not the only phenyl triazenes isolated from our initial screen. LK36, LK37, and LK39, which were eliminated from further study because of their potential for antimicrobial effects against P. aeruginosa (MIC < 25 µg/ml), also share the phenyl triazene core (see Fig. S6 in the supplemental material). Each of these compounds have different alkyl groups on the triazene moiety and various substituents on the phenyl ring. Based on the similarity of the scaffold and the relative similarity of their transcriptional responses, we predict that LK35 and LK38 (and probably the other three phenyl triazenes identified as well) are causing DNA damage, particularly by methylating guanine residues. Although P. aeruginosa (*ogt* and *ada*) and C. elegans (*agt-1* and *agt-2*) (79) are able to repair O^6^MeG, the enzymatic activity in both organisms appears to require direct transfer of the methyl group from guanine to the enzyme, meaning that this process could be saturated in the presence of sufficient damage. DNA damage (especially to the germ line) has been shown to activate host defenses (80). Likely the ability of phenyl triazenes to rescue in liquid killing is driven by a combination of their weak antibacterial activity and immunostimulatory activity in the host.

LK56, the final compound analyzed in this study, is a member of a diverse class of bioactive compounds known as thiazolopyrimidines. Compounds in this group are known to have a variety of activities, including anti-inflammatory, anticancer, analgesic, and neuroleptic activity (for example, ritanserine and setoperone are known serotonin antagonists) (81, 82). Given this wide variety of chemical functions, it will be interesting to see whether LK56 is a direct ligand for NHR-49/HNF4 or whether it prompts the production of a ligand derived from the host or the pathogen.

### Conclusion.

Despite the difficulties inherent in identifying biomolecular mechanisms for compounds identified from whole organism phenotypic screens, the advantages of this technique are also clear. Pipelines of antimicrobials that are “easy” to discover have begun to run worryingly dry, and it is becoming more imperative than ever to find new drugs, preferably in new ways. We are cautiously optimistic that immunostimulants will eventually become a valuable addition to the clinician’s toolset. The five compounds described here show some promising signs of having potential use for this purpose, including dose-dependent responses. Two of the compounds also have some protective effect against a broad range of bacterial pathogens (including both Gram-positive and Gram-negative organisms), and they exhibit strong probability of oral bioavailability, which is an important starting characteristic for drug development efforts. They will also serve as useful tools to aid in understanding host-pathogen interactions and the nature of the C. elegans immune system.

## MATERIALS AND METHODS

### Compounds.

LK32 (PubChem CID 5368832) was purchased from Maybridge Ltd.; LK34 (PubChem CID 629830) and LK35 (PubChem CID 3112778) were purchased from Vitas-M Laboratory; LK38 (PubChem CID 826309) was purchased from Asinex, Ltd.; and LK56 (PubChem CID 2492524) was purchased from Enamine, Ltd. All compounds were purchased through the MolPort chemical marketplace.

### *C. elegans* and bacterial strains.

All C. elegans strains were maintained on nematode growth media (NGM) plates seeded with *E. coli* OP50 (83). Eggs were harvested from gravid adults by hypochlorite isolation and allowed to hatch overnight in S Basal. Worms were maintained at 15°C. The following C. elegans strains were used in this study: *glp-4(bn2)* (84), N2 Bristol (wild-type) (83), *nsy-1(ag3)* (65), *sek-1(km4)* (65), TJ356 *zIs356* [*Pdaf-16*::*daf-16a/b-gfp*; *rol-6(su1006)*], AY101 *acIs101*[*pDB09.1(pF35E12.5*::*GFP)*; *pRF4(rol-6(su1006))*] (70), CL2166 *dvIs19*[*pAF15(gst-4p*::*GFP*::*NLS)*] (85), NVK93 *pJY323(Phsp-16.1*::*GFP)*; *pRF4*, SJ4005 *zcIs4* [*hsp-4*::*GFP*] V (86), GR2183 mgIs72 [*rpt-3p*::*GFP + dpy-5*(*+*)] II (87).

For infection assays, P. aeruginosa strain PA14 (88, 89), methicillin-resistant S. aureus strain MRSA131 (90), and E. faecalis strain OG1RF (29) were used. Bacteria were routinely grown in liquid media, comprised of Luria-Bertani (LB) medium for P. aeruginosa, tryptic soy broth (TSB) for S. aureus, or brain heart infusion broth (BHI) for E. faecalis.

MIC assays to determine the concentration of the compound necessary to prevent bacterial growth were performed in 384-well plates containing the media inoculated with the bacterial strains of interest and the compounds of interest, which had been serially diluted 2-fold. Plates were allowed to grow for 24 h at 37°C without shaking. Growth was scored visually on the basis of turbidity. At least three biological replicates were performed.

### *P. aeruginosa* liquid killing assay.

Liquid killing of C. elegans was performed as previously described with some changes (91). In brief, an overnight culture of LB medium was inoculated with a single colony of *Pseudomonas aeruginosa* strain PA14. Then, 400 µl of culture was spread onto a 10-cm slow-kill agar plate (91) and grown for 24 h at 37°C, followed by 24 h at 25°C. Bacteria were scraped from the plate, resuspended in S Basal medium, and added to 384-well plates with small molecules dissolved in DMSO. Solution in wells contained a final concentration of 40% SK media, 59% S Basal medium, 1% DMSO, and small molecules. For all liquid-based killing assays, solvent control wells contained 1% DMSO. MgSO_4_ and CaCl_2_ (300 µM) and cholesterol (1.6 µg/ml) were added to aid in production of virulence factors. Worms were sorted into 384-well plates using a COPAS FlowSort (Union Biometrica, Holliston, MA), incubated at 25°C until the DMSO control was roughly 35 to 55% dead, washed four times using an EL406 microplate washer (BioTek, VT), and stained with 50 μl of Sytox Orange at 1 µM to fluorescently stain dead worms. Wells were imaged in bright-field and RFP channels using the BioTek Cytation5, followed by image analysis using the CellProfiler software package to calculate fraction of dead worms in each well. EC values were defined as the minimum concentration that provided statistically significant rescue. At least three biological replicates were performed.

### *S. aureus* infection assay.

The S. aureus killing assay was performed as previously described with some changes (26). A single colony was used to inoculate a 5-ml aerobic culture of TSB. The following day, 100 µl of this culture was used to inoculate a second 10 ml of TSB culture wrapped with parafilm. Then, 384-well plates were made containing 10% TSB, and S. aureus from the second culture was resuspended in S Basal medium to a final OD of 0.04. Compounds were then serially diluted in a DMSO-S Basal solution to achieve the desired concentration in the wells with a 1% DMSO final concentration for all compounds. After 5 days, the worms were transferred to new plates using a 0.05% Tween solution to prevent S. aureus biofilm background fluorescence from obscuring dead worms. Subsequent washing, staining, imaging, and image analysis were performed identically to *Pseudomonas* liquid killing. EC values were defined as the minimum concentration that provided statistically significant rescue. At least three biological replicates were performed.

### *E. faecalis* infection assay.

The E. faecalis infection assay was performed as previously described (27). A single colony was used to inoculate a 5 ml of BHI liquid culture. After 16 to 24 h, 400 µl of this culture was spread on a BHI agar plate and placed at 37°C for 24 h. Next, 384-well plates were made containing 10% BHI, and E. faecalis was removed from the plate by scraping and resuspended in S Basal medium to a final OD of 0.03. Compounds at the desired concentrations or DMSO (final concentration, 1%) were then added to each well. After 3 days, the worms were transferred to new plates using a 0.05% Tween solution to prevent background fluorescence from E. faecalis biofilm. Subsequent washing, staining, imaging, and image analysis was performed identically to *Pseudomonas* liquid killing. EC values were defined as the minimum concentration that provided statistically significant rescue. At least three biological replicates were performed.

### *P. aeruginosa* slow-killing assay.

Slow-killing (SK) plates were made as previously reported (92). Compounds were added to molten SK agar before pouring plates. After solidifying, 40 µl of PA14 overnight culture was spread on plates before incubating plates at 37°C for 24 h, followed by 25°C for 24 h. 5-fluoro-2′-deoxyuridine (FUDR) (0.1 mg/ml) was dropped on plates 30 min prior to picking worms, to ensure nematode sterility. Fifty to seventy L4-stage, wild-type worms were picked onto plates and scored every 12 h after the first 24 h, until all worms were dead. Three biological replicates were performed.

### Transcriptional reporter assays.

Worms were washed three times prior to incubation with compounds or DMSO control and diluted in S Basal medium supplemented with E. coli OP50 (OD_600_ = 0.08) as a food source. The fluorescence fold increase for *gst-4p*::GPF was taken as the fluorescence of each well and was normalized to the well’s fluorescence at 0 h. *irg-5p*::GFP, *hsp-16.1p*::GFP, *rpt-6p*::GFP, and *hsp-4*::GFP fluorescence was quantified as the fluorescence/worm area. For *gst-4p*::GFP, 100 µM ethanol-solubilized juglone was used as a positive control. Ethanol was also tested at 1% (vol/vol) as a control. For *irg-5p*::GFP, RPW-24 (100 µM) was used as a positive control. For quantification of nuclear localization of GFP fusion reporters, worms were scored as either localization positive or localization negative. DAF-16::GFP worms were scored as localization positive if >5 nuclei within the worm had localized GFP. All worms were imaged at the L4-young adult stage. At least three biological replicates were performed.

### DHE staining.

Worms were grown to young adults and washed three times with S Basal medium before incubation with LK molecules or DMSO control for 10 h. Worms were then washed and stained with DHE at 4 µM for 1 h before washing them again and measuring the fluorescence/time of flight with a COPAS FlowSort (Union Biometrica). At least three biological replicates were performed.

### Longevity assays.

Worms were picked onto agar plates seeded with 50 µl of concentrated E. coli OP50. Compounds were added to liquid agar before pouring plates. Worms were scored every day for death by prodding with a platinum wire. Worms that escaped the plate or died on the wall of the plate were censored. Each compound was tested in at least three biological replicates, each including 50 to 70 worms.

### MTT assays.

RWPE-1 cells were seeded at 15,000 cells/well and incubated in KSFM complete medium at 37°C for 24 h to allow for attachment. The medium was aspirated and replaced with compounds in KSFM complete medium and held at 37°C for 24 h. The cells were then washed and treated with MTT reagent for 3.5 h. Then, 100 µl of DMSO was added, and the absorbance at 590 nm measured. MTT assays were performed in triplicate.

### nanoString, microarray, gene expression analysis, and gene ontologies.

For nanoString-based (nanoString Technologies, Seattle, WA) experiments, 2,000 wild-type, young adult worms were exposed to 100 μM LK molecules or DMSO control in S Basal medium in 6-well plates for 16 h. RNA purification and gene expression analysis were performed according to nanoString guidelines. Two biological replicates were tested for each condition.

Transcriptome profiling was performed on ∼6,000 wild-type, young adult worms incubated with either LK molecules or DMSO for 8 h. RNA isolation was performed using TRIzol extraction, followed by cleanup using RNeasy columns according to the manufacturer’s protocol. Each condition was analyzed in triplicate. Microarray data have been deposited in the GEO database, accession number GSE137516. Transcriptional profiles of C. elegans treated with LK molecules were used to generate lists of upregulated genes as described previously (93). Genes that were upregulated by at least a factor of 2 were included in the list. Upregulated genes were compared to gene lists dependent upon various defense response pathways and small molecules (15, 39, 47, 52, 94–97) and targets for a wide array of transcription factors (46, 95). Clustering and generation of the heat map was performed using Cluster 3.0 (98) and Treeview (99), respectively. Fold change of genes were normalized to average fold change of that gene over all clustered conditions. Genes and conditions were clustered using Euclidean distance and average linkages. Genes that were not upregulated >2-fold or downregulated <0.5-fold were not included in either upregulated or downregulated gene lists. Microarray data for non-LK molecules were collected from GEO omnibus. WormEXP was used as a secondary method for confirming statistical significance of potential pathways stimulated by each of the LK molecules (100). Gene ontologies were generated using DAVID (101, 102).

### Statistics.

Tanimoto coefficients were calculated using Open Babel (103). A log-rank test was used to determine statistical significance for slow-killing and longevity experiments (http://bioinf.wehi.edu.au/software/russell/logrank/). Student’s *t* test was used to calculate statistical significance for killing assays and GFP reporter strains. Hypergeometric probabilities were calculated using Excel.

### Data availability.

Microarray data have been deposited in the GEO database under accession number GSE137516.
