# Supplementary figures and images for: Novel Immune Modulators Enhance Caenorhabditis elegans Resistance to Multiple Pathogens
Source: mSphere. 2021 Jan 6;6(1):e00950-20. doi: 10.1128/mSphere.00950-20 (PMC7845594; doi:10.1128/mSphere.00950-20)

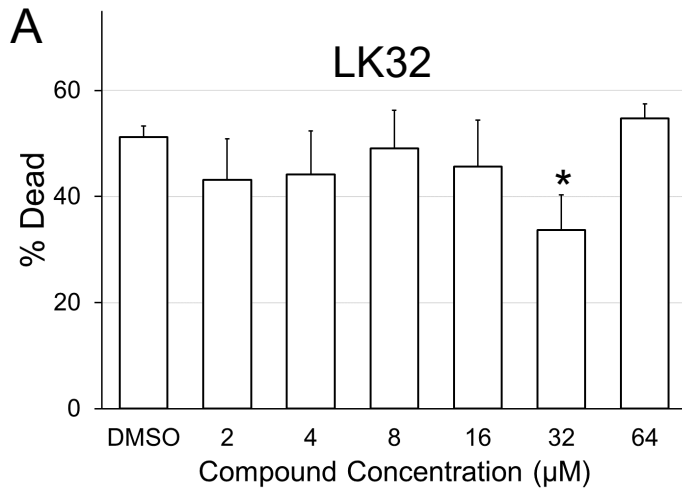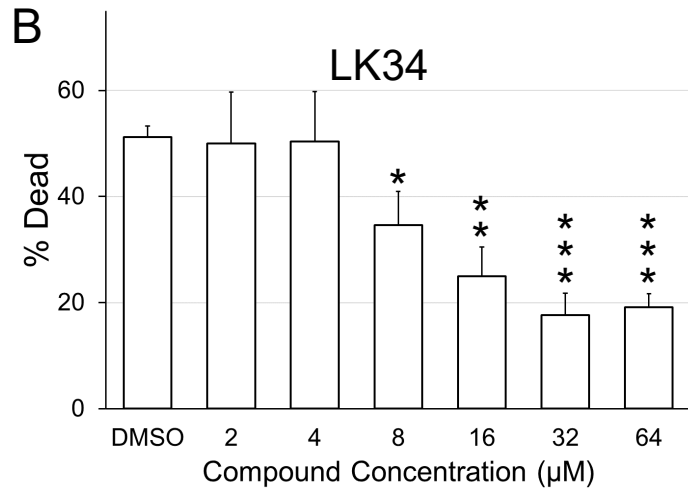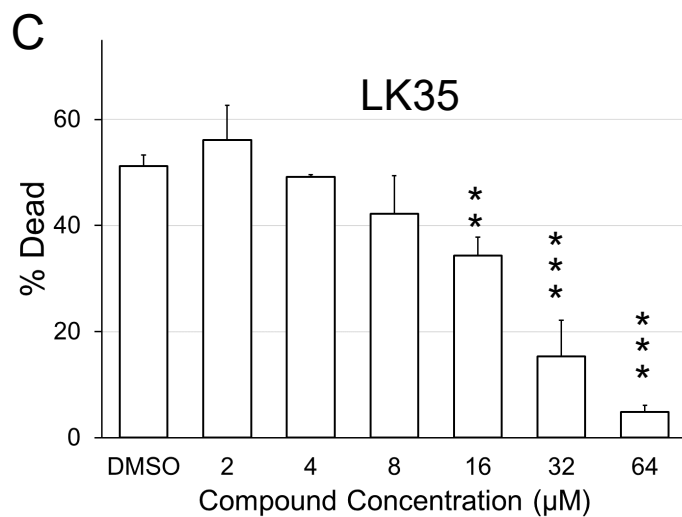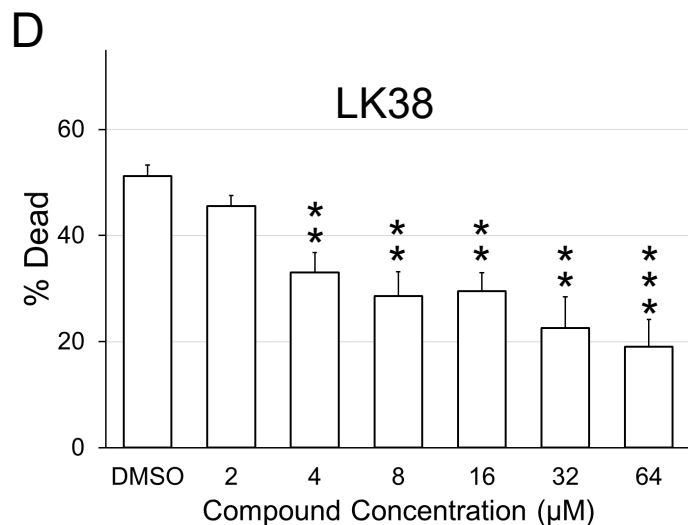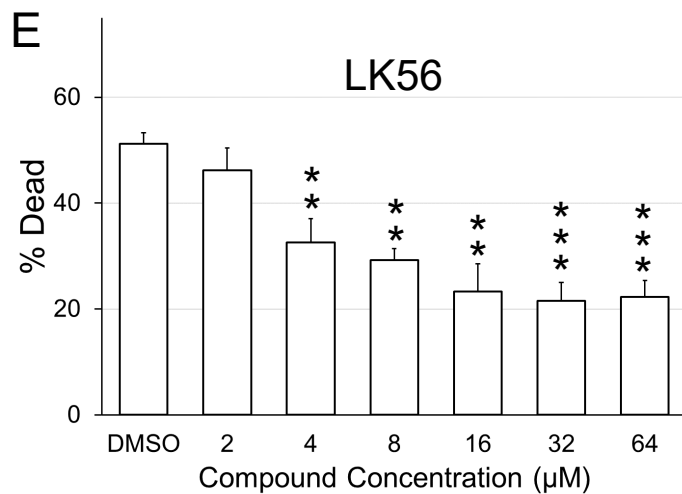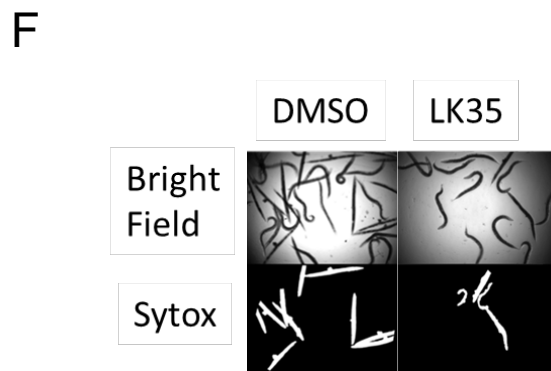

**Figure S1**

Supplement: FIG S1 [file mSphere.00950-20_sf001.pdf]

A

*hsp-16.1p::GFP*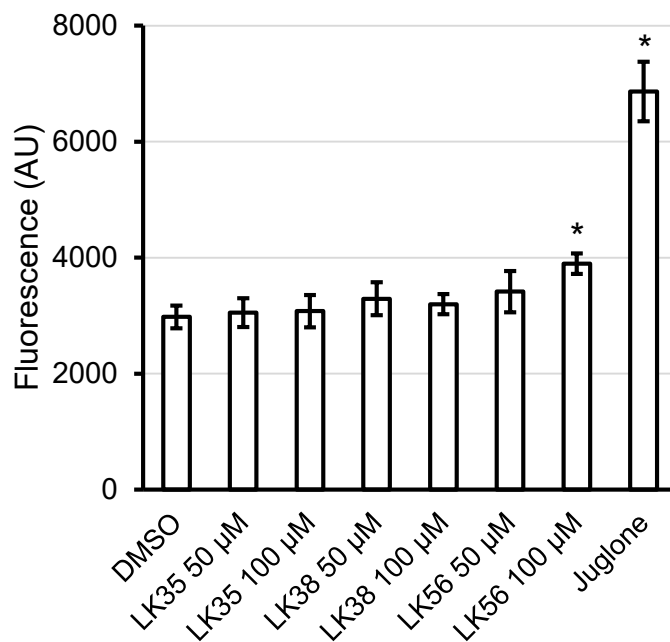

B

DHE staining

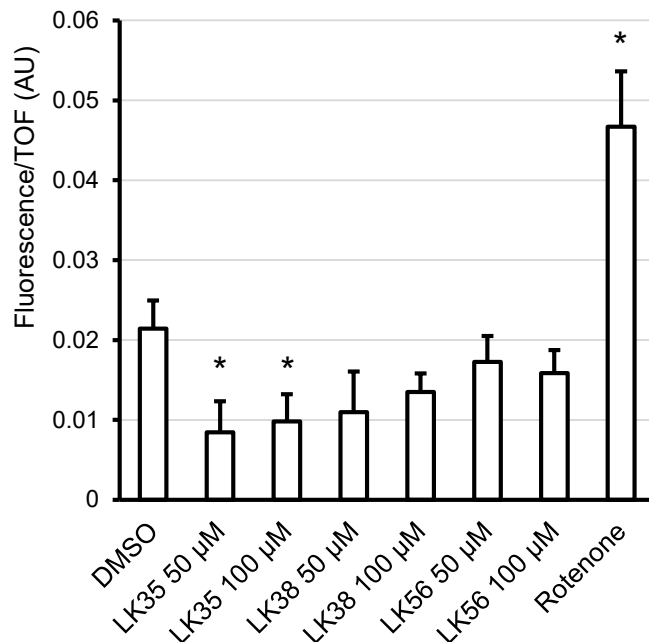

C

*hsp-4p::GFP*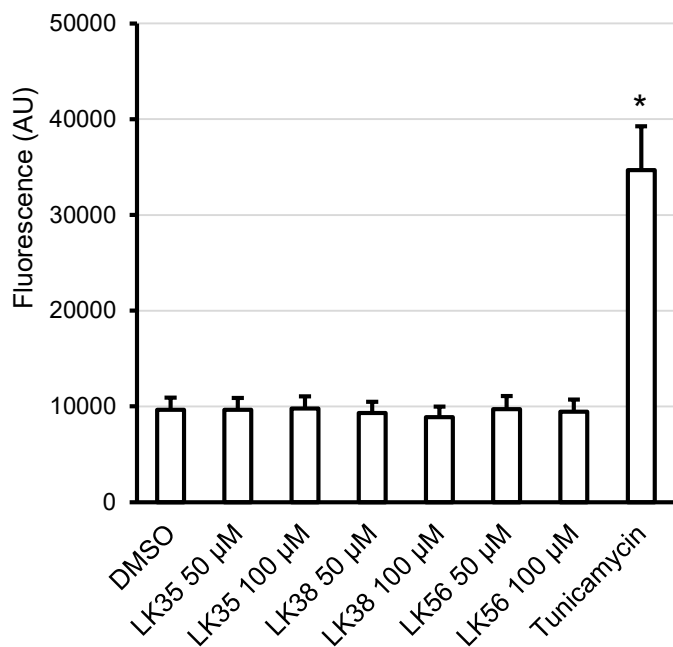

D

*rpt-3p::GPF*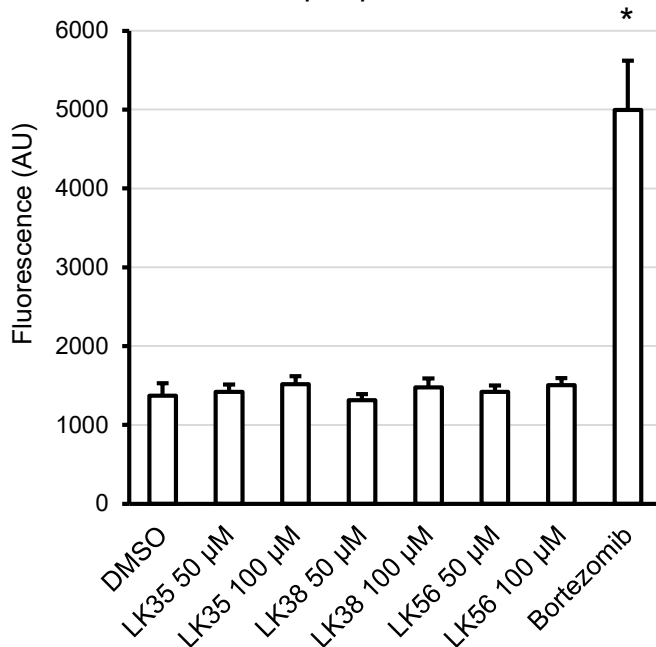

Figure S2

Supplement: FIG S2 [file mSphere.00950-20_sf002.pdf]

**A**

DAF-16 Localization (+)

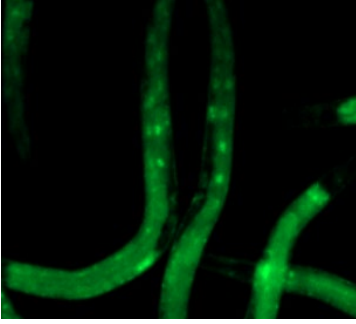**B**

DAF-16 Localization (-)

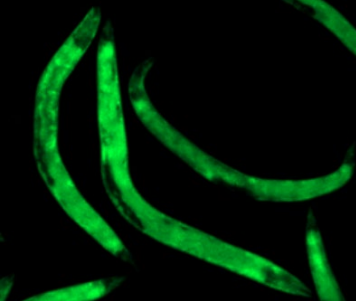**C**

DAF-16::GFP

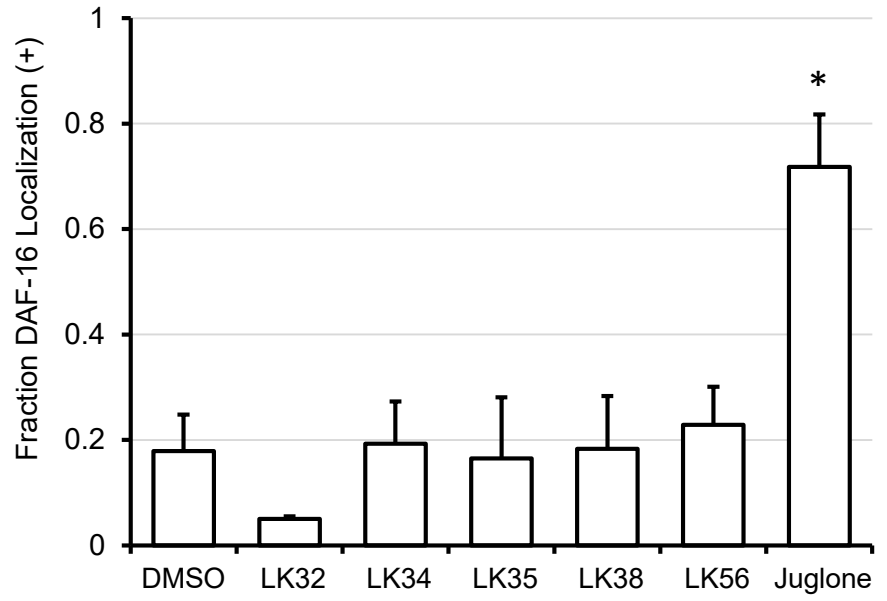**Figure S3**

Supplement: FIG S3 [file mSphere.00950-20_sf003.pdf]

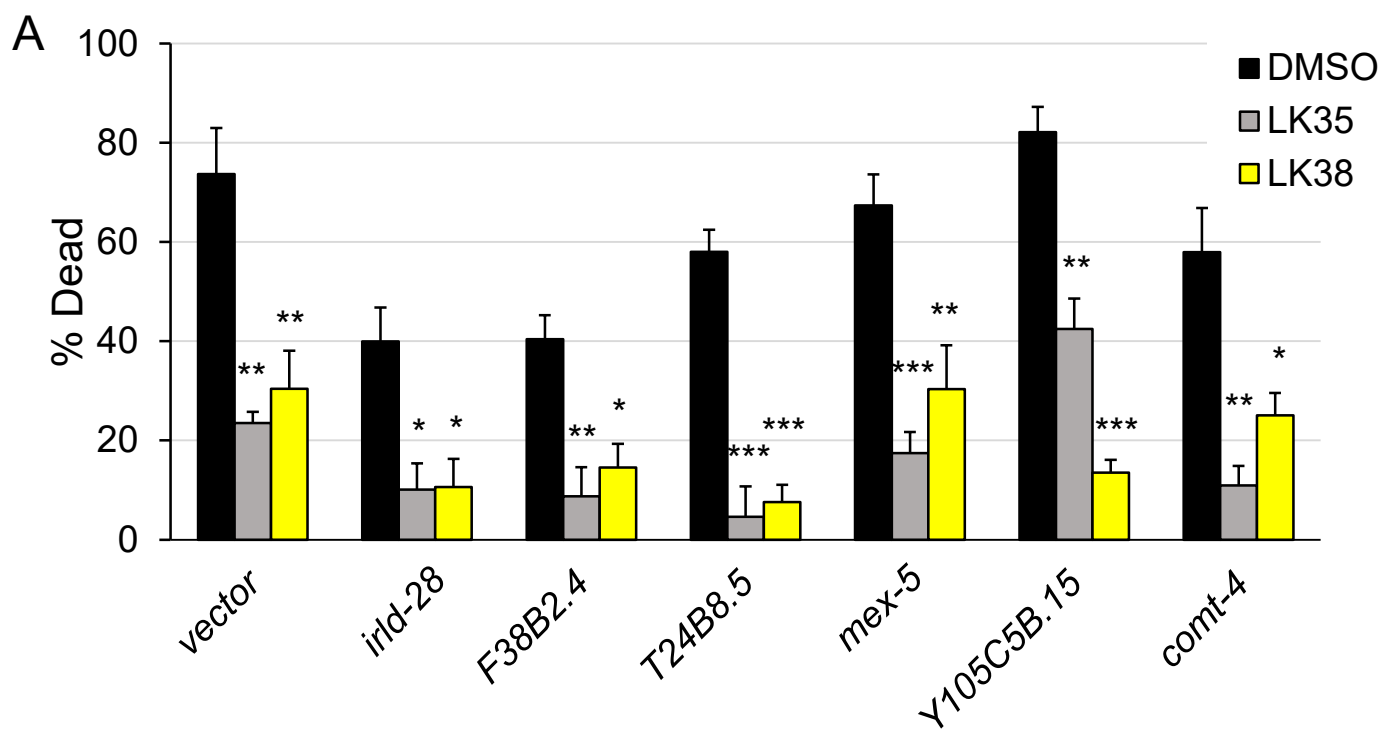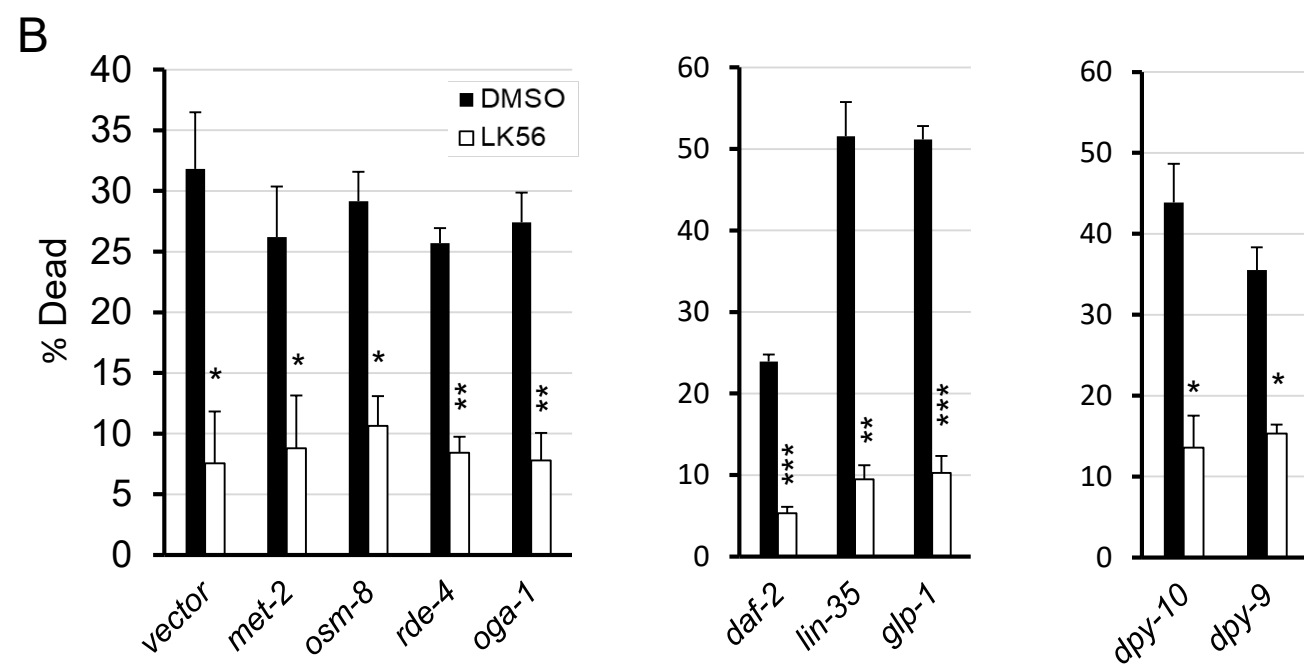

**Figure S4**

Supplement: FIG S4 [file mSphere.00950-20_sf004.pdf]

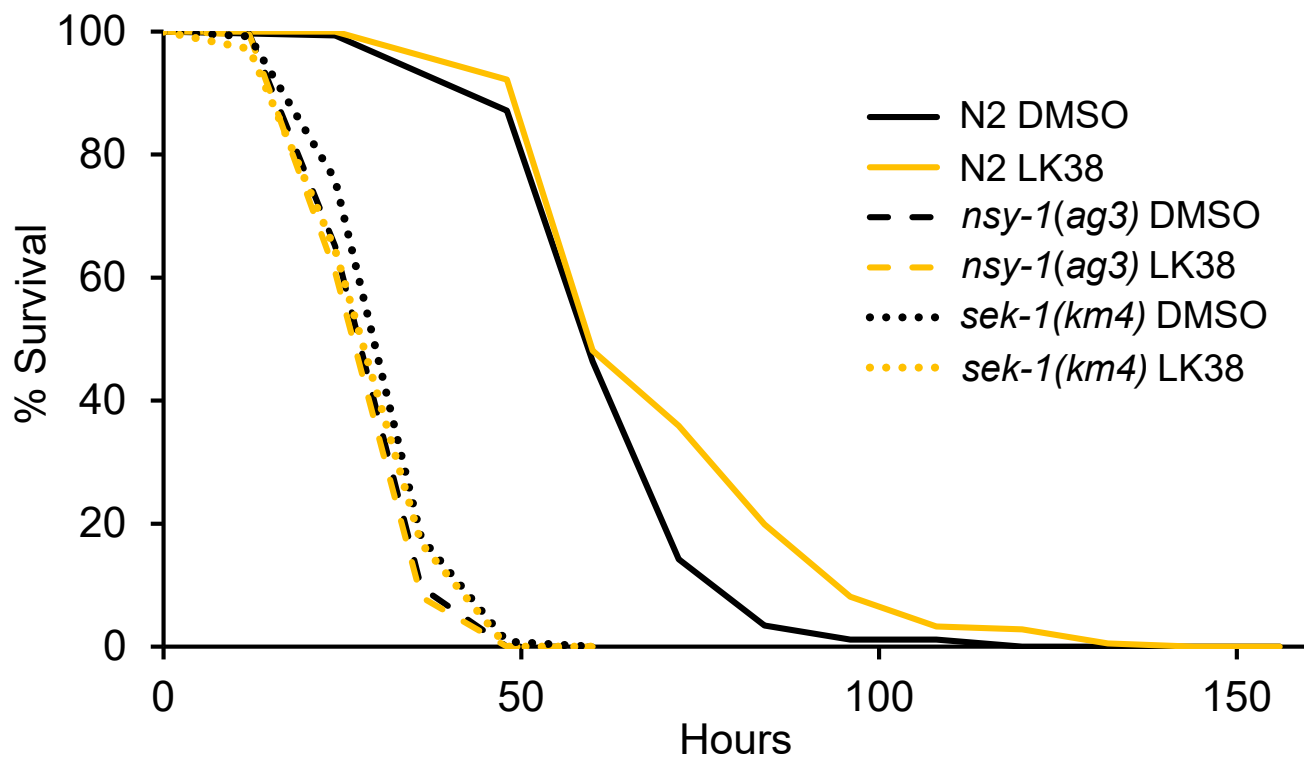

**Figure S5**

Supplement: FIG S5 [file mSphere.00950-20_sf005.pdf]

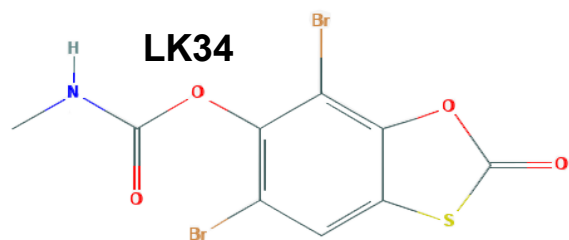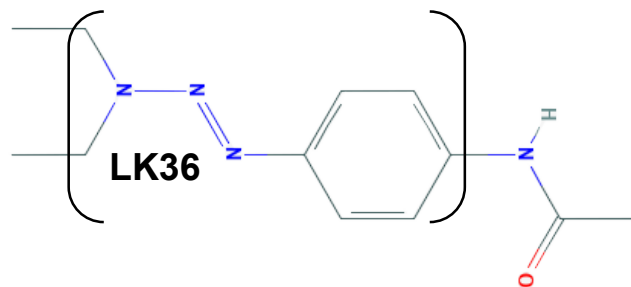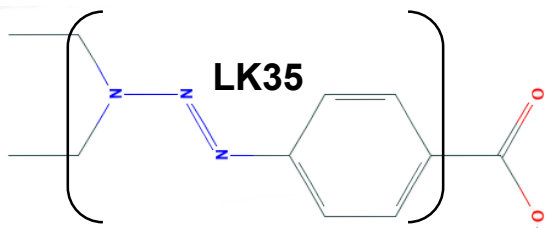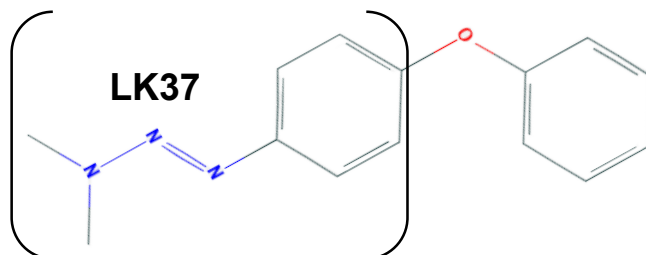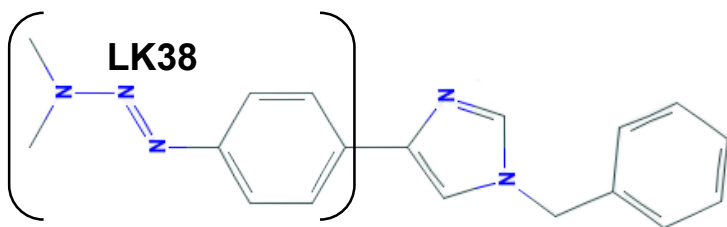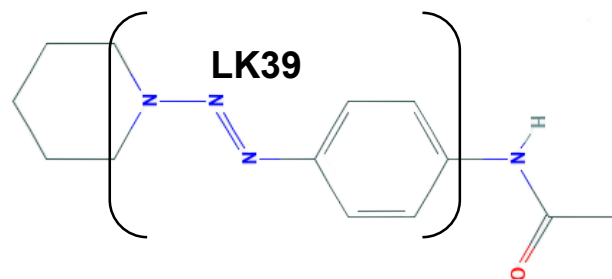

**Figure S6**

Supplement: FIG S6 [file mSphere.00950-20_sf006.pdf]
